# PTH(1–34) treatment and/or mechanical loading have different osteogenic effects on the trabecular and cortical bone in the ovariectomized C57BL/6 mouse

**DOI:** 10.1038/s41598-020-65921-1

**Published:** 2020-06-01

**Authors:** Bryant C. Roberts, Hector M. Arredondo Carrera, Sahand Zanjani-pour, Maya Boudiffa, Ning Wang, Alison Gartland, Enrico Dall’Ara

**Affiliations:** 1 Department of Oncology and Metabolism, University of Sheffield, Sheffield, United Kingdom; 2 Insigneo Institute for in silico Medicine, University of Sheffield, Sheffield, United Kingdom; 3 MRC Arthritis Research UK, Centre for Integrated Research into Musculoskeletal Ageing (CIMA), University of Sheffield, Sheffield, United Kingdom

**Keywords:** Bone, Preclinical research, Therapeutics, Osteoporosis, Three-dimensional imaging

## Abstract

In preclinical mouse models, a synergistic anabolic response to PTH(1–34) and tibia loading was shown. Whether combined treatment improves bone properties with oestrogen deficiency, a cardinal feature of osteoporosis, remains unknown. This study quantified the individual and combined longitudinal effects of PTH(1–34) and loading on the bone morphometric and densitometric properties in ovariectomised mice. C57BL/6 mice were ovariectomised at 14-weeks-old and treated either with injections of PTH(1–34); compressive loading of the right tibia; both interventions concurrently; or both interventions on alternating weeks. Right tibiae were microCT-scanned from 14 until 24-weeks-old. Trabecular metaphyseal and cortical midshaft morphometric properties, and bone mineral content (BMC) in 40 different regions of the tibia were measured. Mice treated only with loading showed the highest trabecular bone volume fraction at week 22. Cortical thickness was higher with co-treatment than in the mice treated with PTH alone. In the mid-diaphysis, increases in BMC were significantly higher with loading than PTH. In ovariectomised mice, the osteogenic benefits of co-treatment on the trabecular bone were lower than loading alone. However, combined interventions had increased, albeit regionally-dependent, benefits to cortical bone. Increased benefits were largest in the mid-diaphysis and postero-laterally, regions subjected to higher strains under compressive loads.

## Introduction

Globally, over 9 million osteoporotic fractures occur annually that may cause permanent disability and increased mortality^1,2^. Annually, the economic burden of osteoporosis (OP) is estimated to exceed €37 billion and $22 billion in the European Union and USA, respectively^3,4^. With an aging population effective treatment strategies are of paramount importance and both pharmacological and non-pharmacological therapies to improve bone properties in OP remain highly sought after^5^.

Recombinant parathyroid hormone [1–34] (PTH(1–34)) is the basis of two FDA-approved bone anabolics prescribed for treatment of OP and associated bone deficits. It is effective for increasing bone mass and strength^6,7^, which clinically corresponded with a 65% and 56% reduction in vertebral^8^ and hip^9^ fractures, respectively. However, high costs and poor treatment adherence are of concern^10^. Alternatively, exercise is also effective in improving bone mineral density (BMD) at both the axial and appendicular skeletal sites^11,12^. While exercise also benefits poor muscle strength and joint mobility that are factors for increased fracture risk, the bony response is reported to be modest at best^12^. PTH and exercise co-therapy may present a promising strategy to alleviate costs and enhance the benefits of each treatment. In a recent randomised controlled trial, PTH with whole body vibration (WBV) produced additional benefits to lumbar vertebra BMD than with PTH treatment alone (8.90% vs. 6.65% increase over 12 months, respectively)^13^. The potential benefits with higher-impact exercise, however, remain unknown.

Preclinical research is useful for the rapid testing of novel anti-osteoporotic therapies, to optimize the osteogenic benefits of different treatment regimens in ovariectomised (OVX) animal models of OP^14^. In rodents and monkeys, PTH reversed OVX-induced trabecular bone loss in the axial^15–18^ and appendicular^16–20^ skeleton. Also, PTH enhanced bone formation at endosteal and periosteal surfaces of the cortical bone when administered over seven weeks either once daily or daily but only on alternate weeks^21^, and increasing the bone strength^17^. Under compressive load or WBV an osteogenic response to mechanical stimuli was also found^15,22–24^. In OVX mice, PTH and loading had increased benefits to the vertebral bone^15^, although in ovary-intact animals, combined treatment had conflicting effects. For example, PTH with passive axial loading or treadmill running has shown increased benefits to both the 3 and 9 months-old rat vertebra^25,26^ and 3–4 months old mouse tibia trabecular bone^27,28^, whereas PTH inhibited the anabolic effect of tibia loading in mature (19 months-old) mice^29^. In cortical bone, both synergistic^27,29^ and neutral effects^30^ with combined treatments were found.

Preclinical assessment of mouse skeletal health most often employs a cross-sectional study design and, in most cases, characterizes bone properties in small regions of interest (e.g. in the tibia metaphysis or cortical midshaft) that underrepresents heterogeneous bone adaptations observed along the limb length. PTH and mechanical loading, for example, affect differently the microarchitecture and densitometric properties in different regions of long bones^29,31^. *In vivo* microCT permits quantification of bone changes, to microns resolution, in the same animal and over time, reducing measurement variability due to inter-subject differences with a considerable reduction in sample size^32,33^. Previous *in vivo* analysis of combined PTH(1–34) and mechanical loading is limited to a single study describing an increased benefit to the trabecular bone in the caudal vertebra of the OVX mouse^15^. However, given there are differences in loading and age-related bone loss in the caudal vertebra compared to anatomical sites which are more physiologically load-bearing, findings may be less translatable to human disease^34^.

The aim of this study was to quantify the longitudinal effects of PTH(1–34) alone, and in combination with mechanical loading, on the bone morphometric and densitometric properties of the tibia in the ovariectomised mouse. The spatiotemporal effects of treatment for four weeks and of treatment withdrawal for two weeks were measured with high-resolution *in vivo* microCT to evaluate detailed early localised changes of the tissue along the bone length.

## Methods

### Animals and treatment

Twenty-four virgin female C57BL/6 mice were purchased at 13-weeks old (Charles River UK Ltd., Margate, UK). Mice were housed, four per cage, in The University of Sheffield’s Biological Services Unit at 22 °C, with a twelve-hour dark/light cycle and ad libitum access to 2918 Teklad Global 18% protein rodent diet (Envigo RMS Ltd., UK) and water. All the procedures were performed under a British Home Office licence (PF61050A3) and in compliance with the Animal (Scientific Procedures) Act 1986. This study was reviewed and approved by the local Research Ethics Committee of The University of Sheffield (Sheffield, UK). The findings and experiments in this paper were designed and reported in accordance with the ARRIVE guidelines^35^. C57BL/6 female mice were chosen due to documented skeletal responsiveness to mechanical loading, PTH(1–34) or OVX^27,29,31,36^. Peak cortical bone mass was reported in the appendicular skeleton of female C57BL/6 mice at 3–4 months of age^37^, thus the mice herein were considered to be skeletally mature at the onset of this study (14 weeks of age). An a priori estimate of sample size based on large loading effects on the trabecular bone volume fraction and cortical thickness after six weeks of loading in PTH-treated mice^27^, indicated that six mice per group was necessary to achieve 80% statistical power and assuming Cohen’s *d* = 2, α = 0.05.

**Figure 1:**
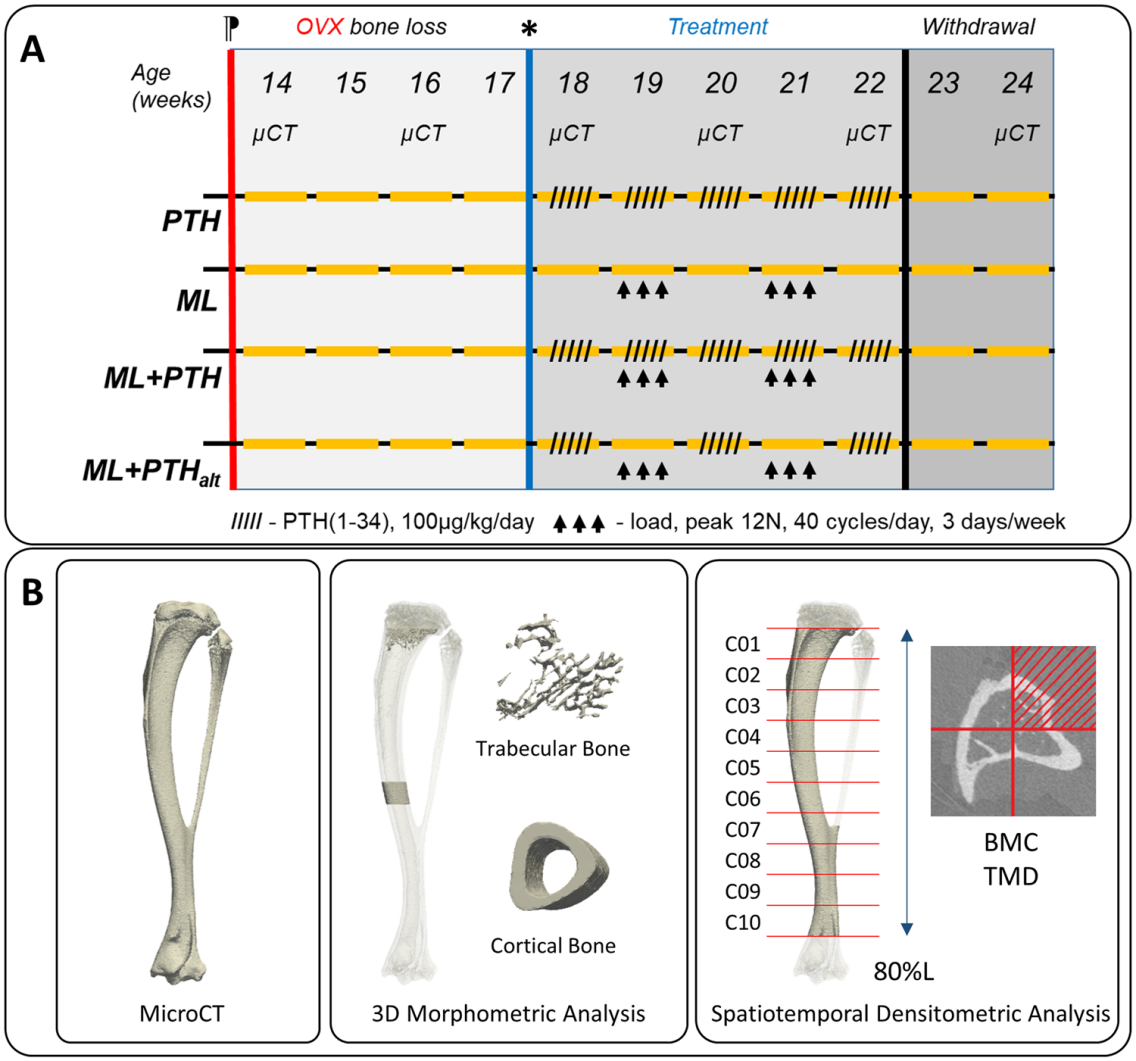
(**A**) Study design and treatment schedule in C57BL/6 mice. ^⁋^Ovariectomy was performed at 14 weeks old; *treatment commenced at age 18 weeks and withdrawn after 22 weeks. Treatment groups (n = 6 mice/group) PTH: PTH(1–34) only; ML: mechanical loading only; ML + PTH: PTH(1–34) and mechanical loading; ML + PTH_alt_: PTH(1–34) and mechanical loading on alternate weeks. (**B**) trabecular and cortical bone regions of interest for (middle) the standard morphometric analysis; and (right) spatiotemporal densitometric analysis.

At age 14 weeks, and following one week acclimatization, all mice underwent OVX and remained untreated for 4 weeks following surgery to allow oestrogen-deficiency related bone loss^36^. OVX mice were randomly assigned into 4 treatment groups (n = 6 mice/group) and then treated, per schedule in Fig. 1(A), with either (1) PTH(1–34) between weeks 18 and 22, subgroup **“PTH”**, (2) mechanical loading during weeks 19 and 21, **“ML”**, (3) concurrent treatment with PTH(1–34) and mechanical loading, **“ML** + **PTH”**, (4) weekly alternating treatment with PTH(1–34) during weeks 18, 20 and 22 of age and mechanical loading during weeks 19 and 21, **“ML** + **PTH**_**alt**_**”**. All mice were withdrawn from treatment for the final two weeks of the study (weeks 23 and 24 of age). We confirmed treatment effects of PTH(1–34) and mechanical loading by comparing bone properties within the same animals before the treatment started (i.e. relative to week 18 values) and with a group of age-matched C57BL/6 ovariectomized mice from a previous study in our laboratory^36^.

### Intraperitoneal PTH(1–34) injections

Mice received either intraperitoneal injection of PTH(1–34) (Bachem, Bubendorf, Switzerland) at 100 µg/kg/day^29,31^, 5 days/week (groups: PTH, ML + PTH) or vehicle (group: ML). PTH was prepared in 1% acetic acid and 2% heat inactivated mouse serum in HBSS^31^.

### Mechanical loading

A minimally invasive method was used for uniaxial compressive loading of the right tibia per a previously published protocol^38^. Briefly, the flexed knee and ankle were fixed between two soft cups and the tibia loaded along the superior-inferior axes to a peak load of 12 N. Tibiae were loaded to 12 N peak by superimposing a dynamic load of 10.0 N upon a static 2.0 N preload at a rate of 160,000 N/second. Forty trapezoidal waveform load cycles were applied (held for 0.2 seconds at 12 N) with a 10 second interval between each cycle. A 12 N load was previously shown to promote significant bone apposition in female C57BL/6 mice without impairing mobility following treatment^38^. Mechanical loading was applied to all mice in groups ML, ML + PTH and ML + PTH_alt_, three days per week (Mon, Wed, Fri) at weeks 19 and 21.

### *In-vivo* microCT imaging

The whole right tibia of each mouse was imaged *in vivo* with microCT (VivaCT80, Scanco Medical, Bruettisellen, Switzerland). A baseline scan (before OVX surgery) was performed at 14 weeks of age, then follow up *in vivo* scans performed every two weeks until week 22 (Fig. 1). At week 24 mice were euthanized by cervical dislocation and both the left and right tibia were imaged *ex vivo* using the *in vivo* imaging protocol. Scanning parameters, optimised in a previous study^39^, were: 10.4 µm isotropic voxel size, voltage of 55 keV, intensity of 145 µA, field of view of 32 mm, 1500/750 samples/projections, integration time 100 ms. This scanning protocol offered the best compromise between image quality and scanning time^39^, which affect both the ionising radiation and the time in which the animal is under anaesthesia. This scanning protocol induced 256 mGy dose to the mouse and was found to have minimal effects on the bone properties evaluated in this study^40^. A third-order polynomial beam hardening correction algorithm based on a 1200 mg HA/cm^3^ wedge phantom was applied to all the scans^41^. A calibration equation based on weekly quality checks performed on a five-rod densitometric phantom was used to convert the Hounsfield Units in each image into tissue mineral density (TMD) equivalent values.

### Image alignment and preprocessing

From each reconstructed microCT image two analyses were performed (Fig. 1(B)): standard 3D morphometric analysis as defined in the guidelines of the American Society of Bone and Mineral Research (ASBMR)^39,42^ and a spatial densitometric analysis^31^. The image of one tibia from one mouse at week 14 of age was randomly chosen and used as a reference. The longitudinal axis of each reference tibia was approximately aligned with the z-axis of the global reference system^43^. All remaining images (from different mice and different time points) were rigidly registered to the reference images prior to the below image analyses. The rigid registrations were performed by using a Quasi-Newton optimizer and the Normalised Mutual Information as the similarity measure (Amira 5.4.3, Thermo Fisher Scientific, France)^44^. The registered grayscale image datasets were smoothed with a Gaussian filter (convolution kernel [3 3 3], standard deviation = 0.65) in order to reduce the high frequency noise and bone voxels were defined using a global threshold, which was calculated as the average of the grey levels corresponding to the bone and background peaks in each image histogram (frequency plot)^39^.

### Standard 3D morphometric analysis

For trabecular bone analysis a region of interest (ROI) of 1 mm height was selected, 0.3 mm below a reference line defined as the most distal image slice that included the growth plate and adapted from previous research^42,45^. This was necessary to minimize analysis of the newly formed (modelled) trabeculae emerging from the growth plate due to continuous longitudinal growth in rodents^46^. For cortical bone analysis a region of 1 mm height was selected in the tibia diaphysis and centred at 50% of the tibia bone length^43^. ROIs in the trabecular and cortical bone were manually marked and the following 3D bone parameters were computed (CT Analyser v1.18.4.0, Skyscan-Bruker, Kontich, Belgium): trabecular bone volume fraction (Tb.BV/TV), trabecular thickness (Tb.Th), trabecular separation (Tb.Sp) and trabecular number (Tb.N); cortical total cross-sectional area (Tt.Ar), cortical bone area (Ct.Ar), cortical area fraction (Ct.Ar/Tt.Ar) and cortical thickness (Ct.Th). In the midshaft cortical ROI, minimum (I_min_) and maximum (I_max_) principal moments of inertia, polar moment of inertia (J) and eccentricity (Ecc) were computed.

### Spatiotemporal densitometric analysis

Densitometric properties were estimated in multiple regions within the tibia adapting a previously described procedure^31^. Briefly, the length of each tibia (L) was measured at each time point, computed as the distance between the most proximal and distal bone voxels in the registered image stack, and a region 80% of L was cropped starting from the section below the growth plate (MatLab, 2018a, The MathWorks, Inc. USA). The tibia was divided longitudinally into ten transverse sections (from most proximal, section C01 to most distal, section C10) with the same thicknesses (i.e. 8% of L) and each section was then divided into quadrants (anterior, medial, posterior and lateral sectors) for a total of 40 ROIs across the length of the tibia. Anterior, medial, posterior and lateral compartments were defined by two perpendicular lines passing through the centre of mass of each slice. Bone mineral content (BMC) and tissue mineral density (TMD, mg HA/cm³) were measured in each of the 10 sections and in each of the 40 quadrants. This approach provides a reasonable compromise between the measurement spatial resolution along the tibia length (number of sections) and the densitometric measurement reproducibility, while accounting for small but still present growth of the tibia between weeks 14 and 24 of age^43,47^.

TMD in each voxel was obtained from its grey level by using the calibration curve provided by the manufacturer of the microCT scanner. The BMC was calculated in each voxel as TMD multiplied by the volume of the voxel. The BMC in each compartment was calculated as the sum of BMC in each bone voxel, while TMD in each compartment was defined as the ratio between BMC and the bone volume (BV)^36^.

### Statistics

All morphometric and densitometric properties were tested for assumptions of normality (Shapiro-Wilks test), homogeneity of variance (Levene’s Test) and sphericity (Mauchly’s Test). To determine whether anabolic treatments reverse OVX-induced trabecular bone loss and cortical bone adaptations, data were analysed by two-way mixed Analysis of Variance (ANOVA). Where for a given bone property the F values were significant for a ‘time by intervention’ interaction, the simple “time effect” was investigated using paired t-tests between (1) treatment baseline (week 18) and proceeding time-points (week 20–24) and (2) between sequential time-points (e.g. week 20–22, 22–24 comparisons)^36,48^. Between-group differences in bone properties due to treatment and treatment withdrawal (i.e. at weeks 20–24) were analysed using Analysis of Covariance (ANCOVA), adjusted for values at 18-weeks-old (treatment onset) and with post hoc pairwise comparisons (Bonferroni-adjusted for six comparisons among treatment groups). Adjustment for week 18 values mitigates bias due to potential differences in the bone properties at the onset of treatment. Statistical significance was set at α = 0.05. All analyses were performed using SPSS Statistics 25 (IBM Corp., Armonk, NY, USA).

Data are presented as mean ± standard deviation (SD) unless otherwise specified. The percentage change in morphometric properties were computed per Eq. (1), where “BP” is the mean bone property value (e.g. of Tb.BV/TV, Ct.Th) and “*i”* defines a subsequent time point (weeks 20–24):

1 $$Relative( \% )change=\frac{(B{P}_{i}-B{P}_{18})}{B{P}_{18}}\times 100 \% $$

Changes of tibia densitometric properties are presented as the mean relative percentage difference between the two treatment groups, normalized for the baseline values of the second group (week 14). See the Supplementary Materials for computation of *mean relative percentage difference* as per Lu *et al*.^31^.

## Results

All mice completed this study without complications. One mouse in the ML + PTH_alt_ group was removed from densitometric analysis as reconstruction of the image data in the distal tibia failed at baseline, but this did not affect morphometric analysis. Data collected in this study are accessible at 10.15131/shef.data.12292787.

### Effects of treatments and withdrawal on the trabecular and cortical bone morphometry

**Figure 2:**
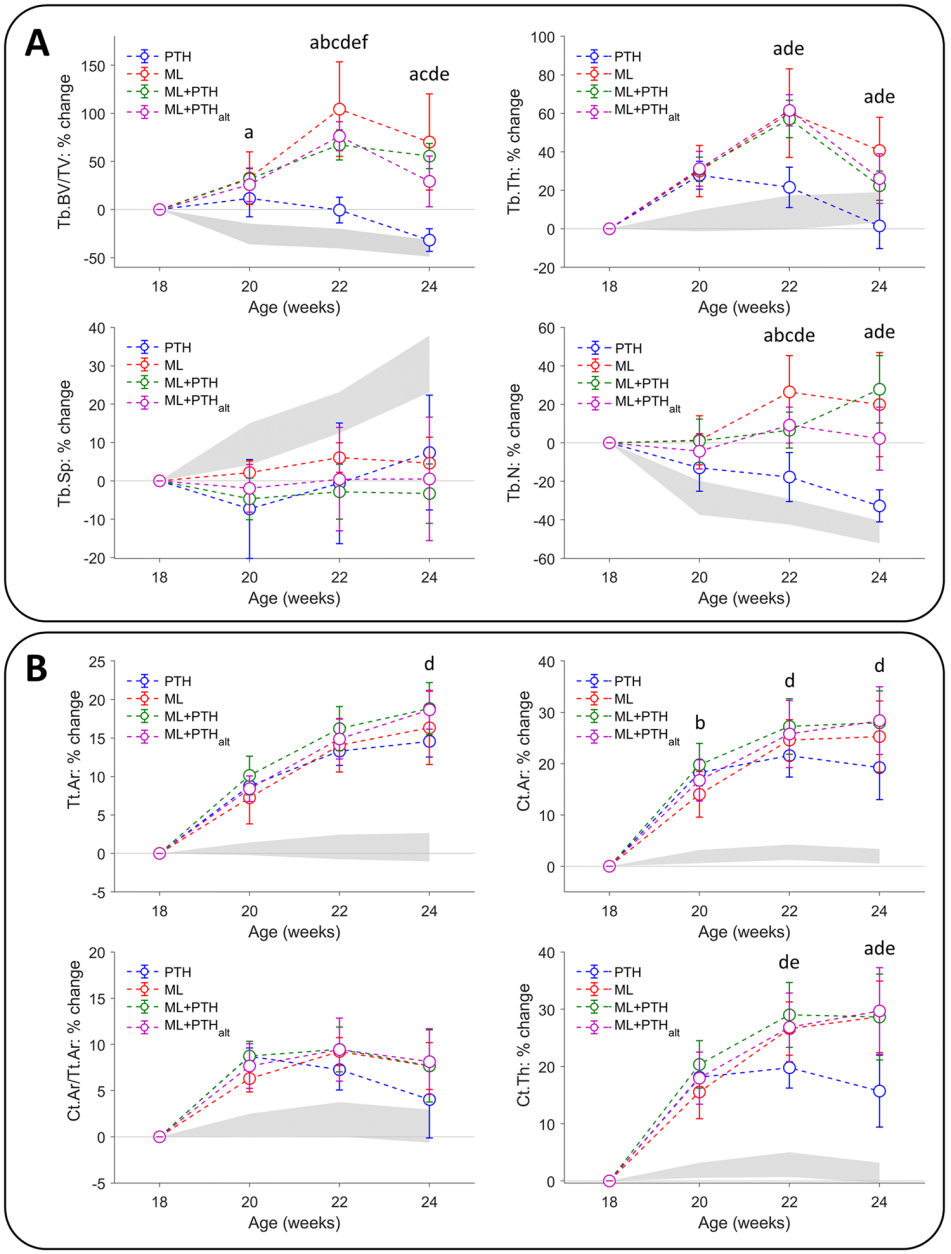
Mean percentage change in (top) trabecular and (bottom) cortical bone 3D morphometric properties in the four treatment groups, relative week 18 values. Treatment commenced at 18 weeks old and was withdrawn at 22 weeks old. Statistically significant differences between groups are noted (p < 0.05; ANCOVA, adjusted for week 18 values with *post hoc* Bonferroni adjustment): ^a^ML vs. PTH; ^b^ML vs. ML + PTH; ^c^ML vs. ML + PTH_alt_; ^d^PTH vs. ML + PTH; ^e^PTH vs. ML + PTH_alt_; ^f^ML + PTH vs. ML + PTH_alt_. Data from untreated ovariectomized mice from a previous study in our laboratory is shown in grey band (±1 SD) highlighting the marked treatment effects of individual and combined treatment with PTH(1–34) and loading on the bone morphometry in C57BL/6 mice.

**Figure 3:**
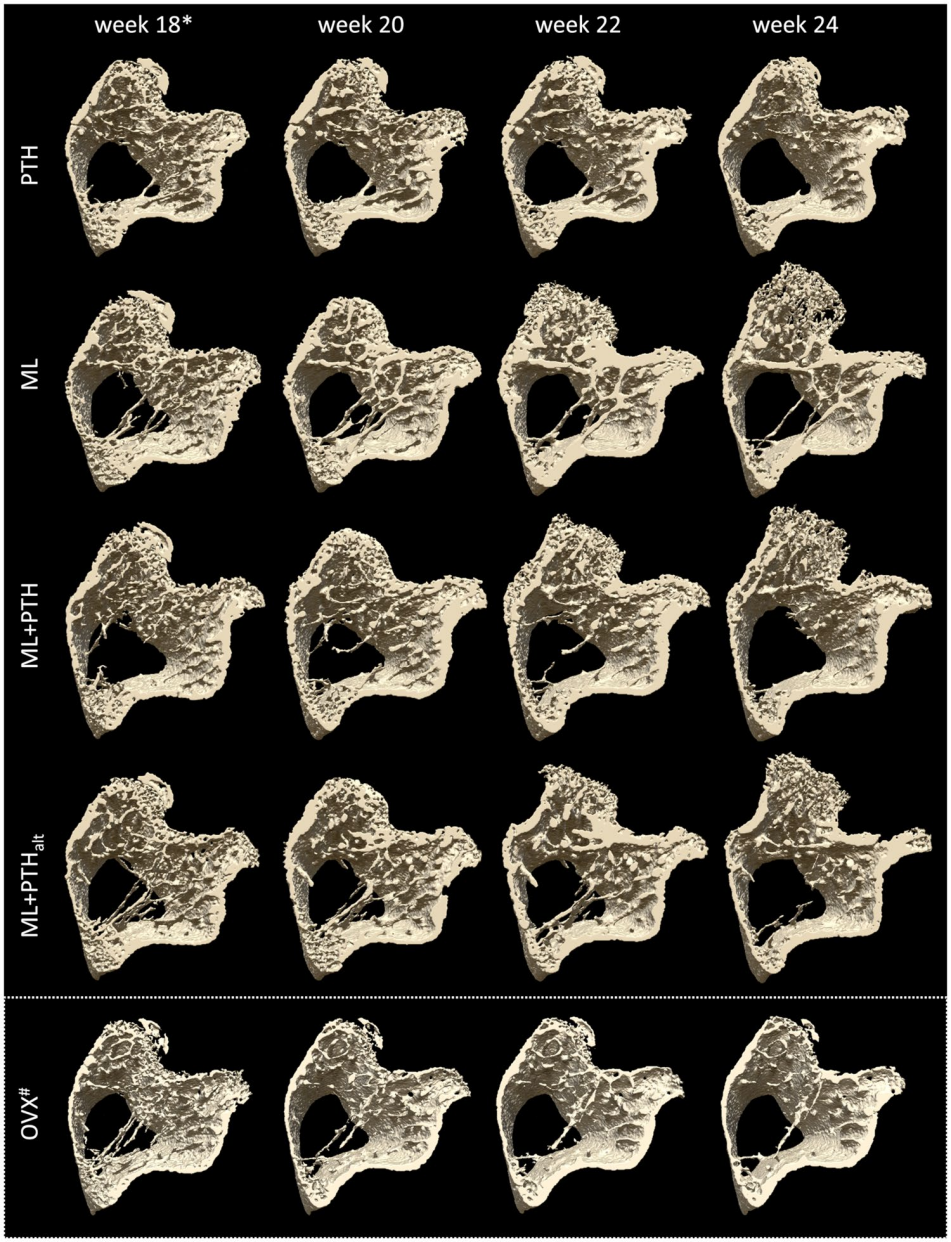
Trabecular bone in the tibia metaphysis of representative mice from the four treatment groups. Figures highlight bony response to individual or combined treatment with PTH(1–34) and mechanical loading in ovariectomized C57BL/6 mice. *Treatment onset commenced at week 18 and was withdrawn after week 22. ^#^OVX changes from an untreated mouse in Roberts *et al*.^36^.

**Figure 4:**
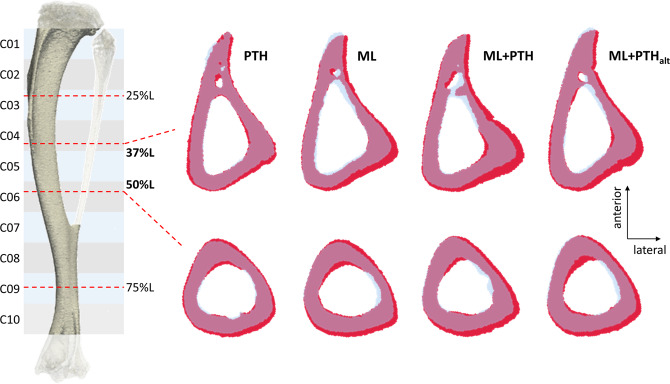
Cortical bone cross-sections at (top row) 37% and (bottom row) 50% of tibia length (L) at 18 weeks old (treatment onset; purple-blue) and 22 weeks old (red). The 37% and 50% reference lines correspond with a bone cross-section in C04 and C06 of the tibia, respectively.

A significant “time by intervention” interaction and “time effect” was observed for all trabecular and cortical morphometric parameters. Thus, the patterns of bone changes in response to anabolic treatment differed among the groups (Figs. 2, 3 and 4). Individual trends for bone morphometric properties are reported in the Supplementary Materials (Fig. S1 and S2).

At week 18 a significant reduction in Tb.BV/TV and Tb.N, and increase in Tb.Sp, relative to week 14 baseline was observed in all groups (Table 1, p < 0.05), and consistent with OVX-induced patterns of bone loss observed previously in C57BL/6 mice (See Supplementary Materials, Fig. S3 and Roberts *et al*.^36^). In PTH, a small and transient, albeit non-statistically significant, increase in Tb.BV/TV was observed at week 20 (12% increase relative to week 18) corresponding with a significant increase in Tb.Th (+27%, p=0.002). Tb.BV/TV returned to baseline at week 22 (2% reduction relative to week 18). In ML, ML + PTH and ML + PTH_alt_, Tb.BV/TV values were significantly higher at week 22 than week 18 (+66–89%, p < 0.01), attributed to a significant increase in Tb.Th (+57–62%, p < 0.02). Individual or combined treatments with mechanical loading did not improve Tb.Sp nor Tb.N, relative to week 18 values. In cortical bone, a detectable (significant) change in morphometric properties was observed within two weeks from treatment onset (week 20). With loading (individually or combined with PTH), a significant and persistent increase in Ct.Ar and Ct.Th was observed from weeks 18 to 20 to 22 (6–20% increase/week, p < 0.01), but only from weeks 18 to 20 with PTH alone (+17–18%, p < 0.001).

**Table 1:** Tibial metaphyseal trabecular 3D bone morphometry over time for each treatment group with ovariectomy (values reported as mean ± standard deviation).

	Treatment	Age (weeks)					
		14^⁋^	16	18*	20	22	24
BV/TV (%)	PTH	7.07 ± 1.22	5.61 ± 1.02	3.70 ± 0.47	4.13 ± 0.85	3.64 ± 0.37	2.51 ± 0.46
	ML	8.60 ± 1.79	6.26 ± 1.58	4.76 ± 1.85	5.97 ± 1.13	9.02 ± 0.86	7.54 ± 1.48
	ML + PTH	7.38 ± 0.99	5.79 ± 0.97	3.87 ± 0.45	5.08 ± 0.48	6.43 ± 0.47	6.01 ± 0.76
	ML + PTH_alt_	8.27 ± 0.87	6.11 ± 0.97	4.51 ± 0.20	5.66 ± 0.73	7.92 ± 0.56	5.80 ± 1.06
	OVX^‡^	6.27 ± 1.19	5.56 ± 1.18	4.26 ± 0.91	3.11 ± 0.43	2.92 ± 0.53	2.49 ± 0.30
	CTRL^‡^	6.75 ± 1.13	6.53 ± 0.79	6.00 ± 0.80	5.72 ± 0.99	5.28 ± 0.53	4.50 ± 0.85
	Time-effect: PTH ML ML + PTH ML + PTH_alt_				(1.000) (0.584) (**0.005**) (0.238)	(1.000;1.000) (**0.005**;**0.001**) (**0.001**;0.086) (**0.001**;**0.003**)	(**0.033**;**0.016**) (0.131;0.631) (**0.002**; 1.000) (0.613;**0.024**)
Tb.Th (µm)	PTH	47 ± 2	46 ± 2	48 ± 2	61 ± 1	58 ± 5	48 ± 4
	ML	47 ± 3	44 ± 4	46 ± 3	59 ± 5	73 ± 7	64 ± 5
	ML + PTH	50 ± 3	48 ± 2	47 ± 4	62 ± 5	74 ± 7	58 ± 4
	ML + PTH_alt_	48 ± 2	45 ± 3	47 ± 4	62 ± 4	76 ± 5	59 ± 4
	OVX^‡^	45 ± 3	42 ± 2	44 ± 3	46 ± 3	48 ± 6	49 ± 6
	CTRL^‡^	45 ± 5	47 ± 5	49 ± 4	52 ± 4	52 ± 5	52 ± 4
	Time-effect: PTH ML ML + PTH ML + PTH_alt_				(**0.002**) (**0.028**) (**0.001**) (**0.002**)	(0.053;1.000) (**0.011**;**0.045**) (<**0.001**;**0.016**) (<**0.001**;**<0.001**)	(1.000;**0.017**) (**0.022**;0.090) (**0.010**;**0.006**) (**0.032**;**0.003**)
Tb.Sp (µm)	PTH	273 ± 21	336 ± 48	449 ± 46^**+**^	416 ± 73	445 ± 78	481 ± 74
	ML	258 ± 29	299 ± 46	362 ± 54^**+**^	369 ± 53	385 ± 66	380 ± 73
	ML + PTH	292 ± 43	342 ± 30	420 ± 56	398 ± 44	405 ± 37	403 ± 40
	ML + PTH_alt_	271 ± 16	321 ± 12	385 ± 46^**+**^	375 ± 29	383 ± 35	382 ± 38
	OVX^‡^	331 ± 29	390 ± 35	411 ± 64	450 ± 75	483 ± 73	535 ± 86
	CTRL^‡^	325 ± 58	342 ± 61	362 ± 62	364 ± 55	388 ± 78	409 ± 70
	Time-effect: PTH ML ML + PTH ML + PTH_alt_				(1.000) (1.000) (1.000) (1.000)	(1.000;0.630) (0.283;1.000) (1.000;1.000) (1.000;1.000)	(1.000;**0.007**) (1.000;1.000) (1.000;1.000) (1.000;1.000)
Tb.N 1/mm)	PTH	1.5 ± 0.2	1.2 ± 0.2	0.8 ± 0.1	0.7 ± 0.1	0.6 ± 0.1	0.5 ± 0.1
	ML	1.8 ± 0.4	1.4 ± 0.4	1.0 ± 0.4	1.0 ± 0.3	1.3 ± 0.2	1.2 ± 0.3
	ML + PTH	1.5 ± 0.2	1.2 ± 0.2	0.8 ± 0.1	0.8 ± 0.1	0.9 ± 0.1	1.0 ± 0.1
	ML + PTH_alt_	1.7 ± 0.2	1.4 ± 0.2	1.0 ± 0.1	0.9 ± 0.1	1.0 ± 0.1	1.0 ± 0.2
	OVX^‡^	1.4 ± 0.2	1.3 ± 0.3	1.0 ± 0.2	0.7 ± 0.1	0.6 ± 0.1	0.5 ± 0.1
	CTRL^‡^	1.5 ± 0.3	1.4 ± 0.2	1.2 ± 0.2	1.1 ± 0.2	1.0 ± 0.3	0.9 ± 0.2
	Time-effect: PTH ML ML + PTH ML + PTH_alt_				(0.644) (1.000) (1.000) (1.000)	(0.362;1.000) (0.375;**0.017**) (1.000;1.000) (1.000;0.109)	(**0.004**;0.227) (1.000;1.000) (0.100;0.176) (1.000;1.000)

BV/TV: trabecular bone volume fraction, Tb.Th: trabecular thickness, Tb.Sp: trabecular separation, Tb.N: trabecular number. The p-values for a “time-effect” are reported in parentheses as (comparison to baseline (week 18) values; comparison previous time point). Bold values indicate a statistically significant difference between time points. Superscript: ^⁋^Ovariectomy was performed at week 14; *treatment commenced, per Fig. 1(a), at the beginning of week 18 and was withdrawn at the end of week 22. +Tb.Sp was significantly higher in PTH than in ML (p=0.013) and ML + PTHalt (p=0.036) at the onset of treatment. Remaining morphometric parameters did not significantly differ among the four treatment groups at week 18 following randomisation. ^‡^3D morphometry of untreated ovariectomized mice (group “OVX”) and intact controls (“CTRL”) from Roberts *et al*.^36^ are reported for comparison of trends in bone adaptation.

Significant intervention effects were observed among treatment groups (Fig. 2). At week 22, Tb.BV/TV, adjusted for week 18 values, significantly differed among all treatment groups and was higher in ML, ML + PTH and ML + PTH_alt_ than PTH (76–148% higher, p < 0.05), and higher in ML than ML + PTH and ML + PTH_alt_ (14–60%, p < 0.05). Tb.Th and Tb.N were significantly lower in PTH than all other groups (−20%, −22%, and −24% for Tb.Th, and −58%, −50%, and −50% for Tb.N compared to ML, ML + PTH, and ML + PTH_alt_, respectively); and Tb.N was 17% lower with combined treatments than ML. In cortical bone, Ct.Th was 14–16% higher with combined treatment than PTH. Ct.Ar was 12% higher in ML + PTH than PTH.

Significant changes in trabecular bone morphometry were observed following treatment withdrawal (Table 1 and Figs. 2 and 3). In PTH, a significant reduction in Tb.BV/TV (31%) and Tb.Th (17%), with corresponding increase in Tb.Sp (8%) was observed between weeks 22 and 24; Tb.BV/TV reductions being similar to change in untreated OVX mice. With combined treatment, significant thinning of the trabecular bone was observed, corresponding with a significant reduction in Tb.BV/TV in ML + PTH_alt_. In ML, changes in trabecular morphometry were not significant following treatment withdrawal. No significant change in cortical bone morphometry (Table 2) was evident between weeks 22 and 24 in PTH, nor ML. In ML + PTH and ML + PTH_alt_, a significant increase in the Tt.Ar persisted following treatment withdrawal.

**Table 2:** Tibial cortical midshaft 3D bone morphometry over time for each treatment group with ovariectomy (values reported as mean ± standard deviation).

		Age (weeks)					
		14^⁋^	16	18*	20	22	24
Tt.Ar (mm^2^)	PTH	0.95 ± 0.03	0.97 ± 0.03	0.96 ± 0.02	1.05 ± 0.03	1.09 ± 0.03	1.10 ± 0.04
	ML	0.91 ± 0.04	0.94 ± 0.03	0.95 ± 0.03	1.02 ± 0.05	1.08 ± 0.04	1.10 ± 0.05
	ML + PTH	0.95 ± 0.03	0.98 ± 0.03	1.00 ± 0.04	1.10 ± 0.03	1.16 ± 0.02	1.18 ± 0.02
	ML + PTH_alt_	0.91 ± 0.05	0.95 ± 0.04	0.96 ± 0.04	1.04 ± 0.04	1.10 ± 0.04	1.14 ± 0.04
	OVX^‡^	0.84 ± 0.03	0.88 ± 0.03	0.89 ± 0.02	0.90 ± 0.03	0.90 ± 0.04	0.90 ± 0.04
	CTRL^‡^	0.83 ± 0.05	0.86 ± 0.04	0.87 ± 0.04	0.88 ± 0.05	0.89 ± 0.04	0.88 ± 0.04
	Time-effect: PTH ML ML + PTH ML + PTH_alt_				(**0.002**) (0.062) (**0.002**) (**0.001**)	(**<0.001**;**0.002**) (**0.001**;**0.006**) (**<0.001**;**0.011**) (**<0.001**;**0.002**)	(**<0.001**;0.875) (**0.005**;0.491) (**<0.001**;**0.022**) (**<0.001**;**0.002**)
Ct.Ar (mm^2^)	PTH	0.58 ± 0.02	0.58 ± 0.02	0.58 ± 0.02	0.68 ± 0.03	0.70 ± 0.03	0.69 ± 0.04
	ML	0.56 ± 0.01	0.57 ± 0.02	0.57 ± 0.01	0.65 ± 0.03	0.71 ± 0.02	0.71 ± 0.04
	ML + PTH	0.58 ± 0.02	0.60 ± 0.02	0.60 ± 0.02	0.72 ± 0.02	0.76 ± 0.01	0.77 ± 0.03
	ML + PTH_alt_	0.56 ± 0.03	0.58 ± 0.03	0.59 ± 0.03	0.69 ± 0.03	0.74 ± 0.03	0.75 ± 0.03
	OVX^‡^	0.50 ± 0.03	0.51 ± 0.03	0.52 ± 0.02	0.53 ± 0.02	0.54 ± 0.02	0.53 ± 0.02
	CTRL^‡^	0.48 ± 0.05	0.51 ± 0.05	0.52 ± 0.05	0.53 ± 0.05	0.54 ± 0.04	0.53 ± 0.04
	Time-effect: PTH ML ML + PTH ML + PTH_alt_				(**<0.001**) (**0.010**) (**0.001**) (**0.002**)	(**0.001**;0.411) (**<0.001**;**0.001**) (**<0.001**;**0.023**) (**0.002**;**0.015**)	(**0.006**;1.000) (**0.006**;1.000) (**0.001**;1.000) (**0.002**;0.162)
Ct.Ar/Tt.Ar (%)	PTH	60.9 ± 0.6	59.9 ± 0.5	59.8 ± 0.6	65.0 ± 0.7	64.1 ± 1.4	62.2 ± 2.4
	ML	61.5 ± 1.4	60.4 ± 1.4	60.3 ± 1.4	64.1 ± 2.2	65.9 ± 2.2	64.9 ± 2.9
	ML + PTH	61.0 ± 0.9	60.9 ± 1.2	60.3 ± 1.7	65.6 ± 1.9	66.0 ± 1.8	64.9 ± 2.5
	ML + PTH_alt_	61.7 ± 0.8	61.3 ± 1.0	61.2 ± 1.1	65.8 ± 1.5	66.9 ± 1.6	66.1 ± 1.5
	OVX^‡^	58.7 ± 0.6	58.5 ± 1.4	58.6 ± 1.4	59.4 ± 1.1	59.7 ± 1.2	59.3 ± 1.4
	CTRL^‡^	57.5 ± 3.3	58.6 ± 3.2	60.0 ± 2.7	60.3 ± 2.7	60.4 ± 2.3	60.3 ± 2.1
	Time-effect: PTH ML ML + PTH ML + PTH_alt_				(**<0.001**) (**0.003**) (**0.001**) (**0.008**)	(**0.007**;1.000) (**0.001**;**0.013**) (**0.003**; 1.000) (**0.015**; 0.500)	(0.962;0.259) (**0.013**;1.000) (0.068;1.000) (**0.036**;0.253)
Ct.Th (µm)	PTH	223 ± 3	221 ± 4	219 ± 4	258 ± 8	262 ± 10	253 ± 13
	ML	222 ± 3	220 ± 6	220 ± 5	255 ± 14	279 ± 14	284 ± 18
	ML + PTH	224 ± 3	226 ± 6	225 ± 7	271 ± 11	290 ± 12	289 ± 17
	ML + PTH_alt_	223 ± 8	226 ± 8	226 ± 9	267 ± 11	287 ± 10	293 ± 9
	OVX^‡^	201 ± 6	205 ± 9	206 ± 8	209 ± 7	211 ± 7	208 ± 8
	CTRL^‡^	196 ± 16	203 ± 16	210 ± 16	212 ± 15	213 ± 13	212 ± 12
	Time-effect: PTH ML ML + PTH ML + PTH_alt_				(**<0.001**) (**0.008**) (**0.001**) (**0.002**)	(**0.001**;1.000) (**0.001**;**<0.001**) (**0.001**;**0.011**) (**0.001**;**0.012**)	(**0.024**;0.835) (**0.002**;1.000) (**0.003**;1.000) (**0.002**;0.706)

Tt.Ar: Total cross-sectional area, Ct.Ar: cortical bone area, Ct.Ar/Tt.Ar: cortical area fraction, Ct.Th: cortical thickness. The P Values for a “time-effect” are reported in parentheses as (comparison to baseline (week 18) values;comparison to previous time point). Bold values indicate a statistically significant difference between time points. ^⁋^Ovariectomy was performed at week 14; *treatment commenced, as per Fig. 1(A), at the beginning of week 18 and was withdrawn at the end of week 22. Morphometric parameters did not significantly differ among the four groups at onset of treatment (p > 0.05). ^‡^3D morphometry of untreated ovariectomized mice (group “OVX”) and intact controls (“CTRL”) from Roberts *et al*.^36^ are reported for comparison of trends in bone adaptation.

With PTH and/or loading, a persistent increase in the cortical midshaft moments of inertia (I_max_, I_min_ and J) and eccentricity were observed (10–49% increase at week 22 compared with week 18 values, p < 0.05), which were retained two-week following treatment withdrawal (Supplementary Table S1). Whereas, in untreated mice, no change in bone properties were observed with ovariectomy over time. At week 22, I_max_ and J were significantly higher in ML + PTH than PTH alone (Fig. S4).

### Effects of treatment on the bone densitometric properties

**Figure 5:**
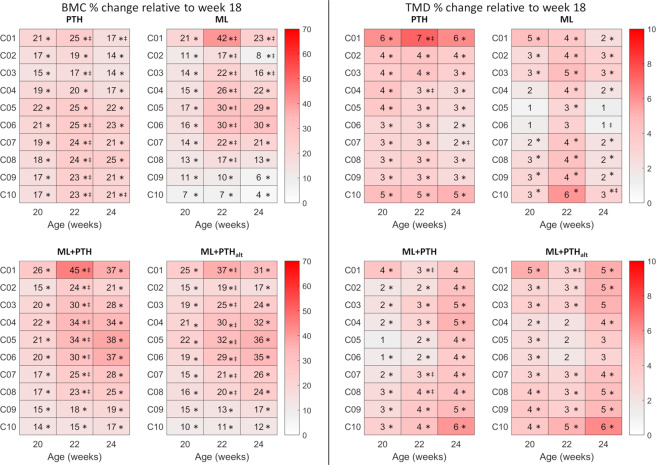
Mean percentage change, relative to week 18 values, in (left) bone mineral content (BMC) and (right) tissue mineral density (TMD) in 10 regions of interest along 80% of the tibia length in the four treatment groups. Sections are C01, most proximal to C10, most distal. Ovariectomy was performed at 14 weeks old and treatment commenced at 18 weeks old and was withdrawn at 22 weeks old. *Statistically significant difference compared with week 18 and ^‡^between sequential timepoints (p < 0.05; ANOVA with post hoc pairwise comparisons).

**Figure 6:**
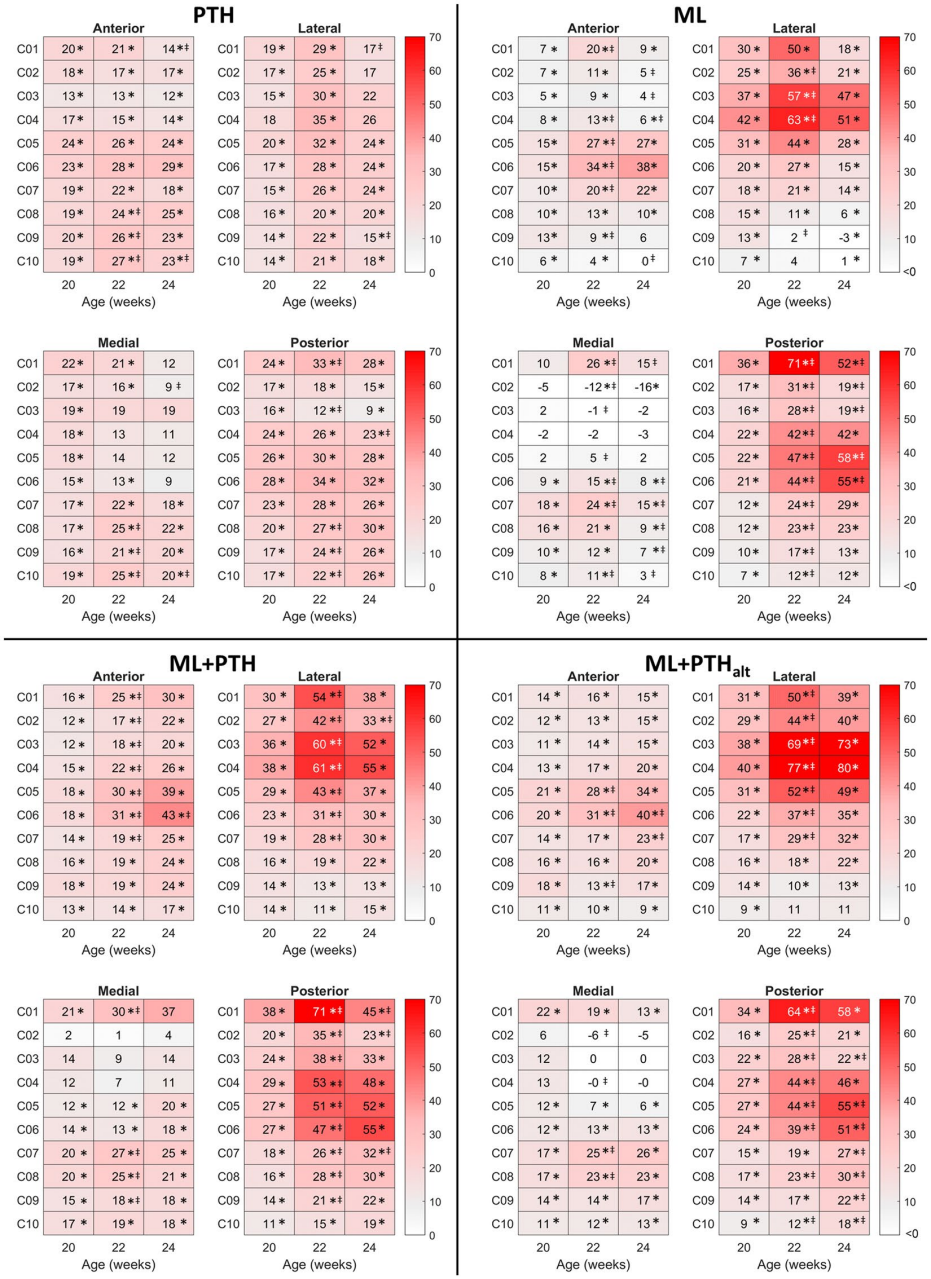
Mean percentage change, relative to week 18 values, in bone mineral content in 40 subregions of interest along 80% of the tibia length in the four treatment groups. Sections are C01, most proximal to C10, most distal. Ovariectomy was performed at 14 weeks old and treatment commenced at 18 weeks old and was withdrawn at 22 weeks old. *Statistically significant difference compared with week 18 and ^‡^between sequential timepoints (p < 0.05; ANOVA with post hoc pairwise comparisons).

At week 20, BMC significantly increased between 7% and 26% along the tibia length in all treatment groups (Fig. 5). With PTH, a significant and persistent increase in BMC, from weeks 18–20–22, were observed in the most proximal (C01, 25% increase from week 18 to 22), the mid (C03, + 17%) and in the distal tibia (C06-C10, + 23 to +25%). In ML and co-treated mice, persistent increments in BMC (from 18 to 20 to 22) were observed in the proximal to mid-tibia (C01-C08, + 17 to +45%). BMC remained above week 18 values following treatment withdrawal. Sub-regionally, the greatest osteogenic benefits of loading and co-treatment were observed posteriorly and laterally, particularly in the most proximal (C01, up to +71%, Fig. 6) and mid-tibia (C03-C06; up to +63%), whereas in PTH a more homogeneous response among quadrants was observed. Every treatment increased slightly the TMD (up to 7% in the most proximal region of the PTH treated mice at week 22 of age) in most regions of the tibia (Fig. 5). While PTH increased the TMD homogeneously across the tibia and among all quadrants, for all loaded mice the central portion of the tibia was less affected particular in the anterior and medial regions (Fig. S6). In most cases small effects on TMD were maintained after treatment withdrawal.

**Figure 7:**
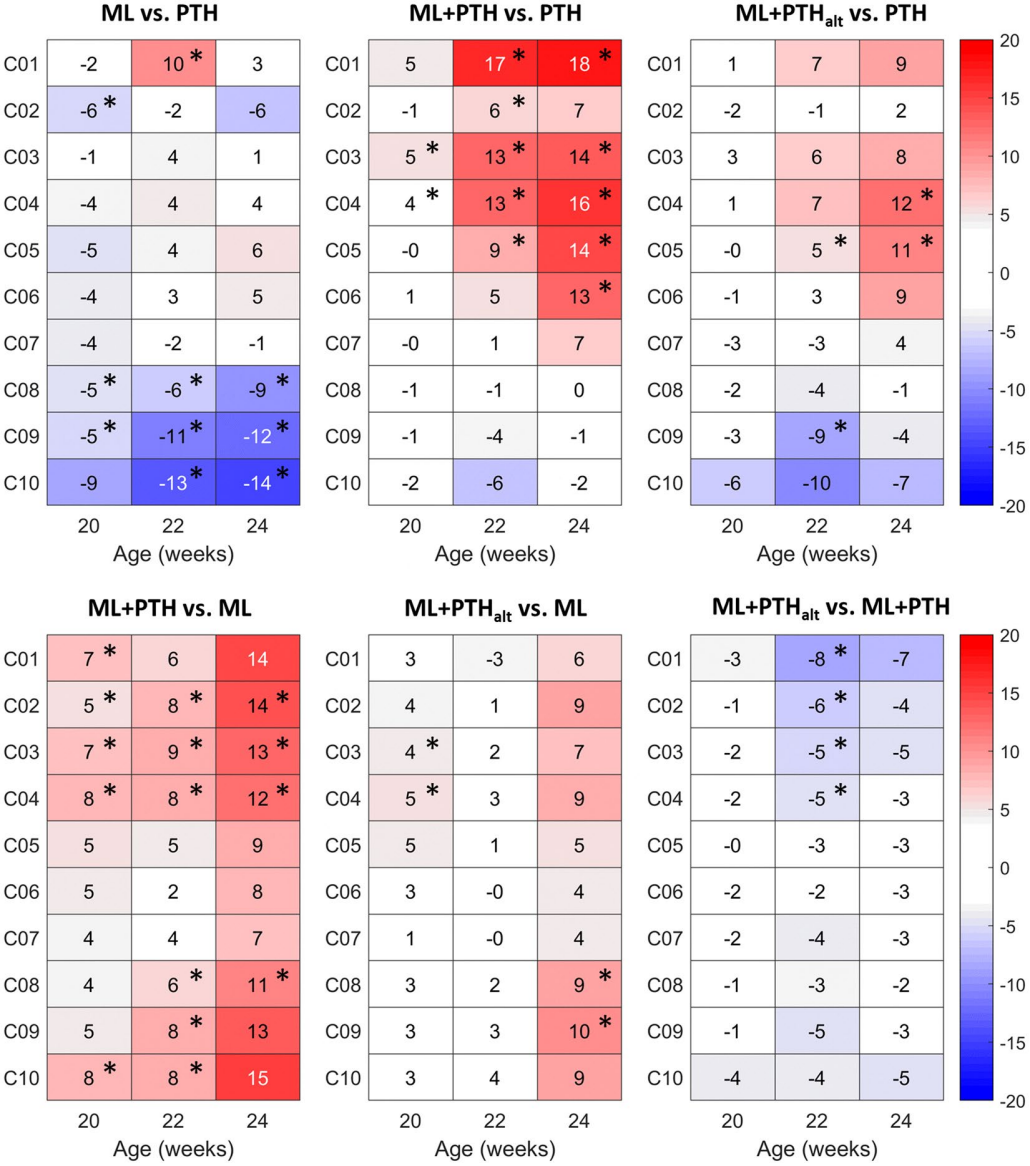
Longitudinal effects of PTH(1–34) and mechanical loading on the bone mineral content (BMC) in 10 sections along the tibia length in ovariectomized C57BL/6 mice. Sections are C01, most proximal to C10, most distal. Values are reported as the relative percentage difference between two treatment groups (*g1* vs. *g2*), normalised for week 18 values of the latter group (*g2*). *p < 0.05, indicates statistically significant differences between groups (ANCOVA, adjusted for baseline values at week 18). Positive and negative values indicate greater increases in BMC in g1 and g2, respectively.

Treatment effects differed among groups (Fig. 7). In ML, the increase in BMC was significantly higher proximally (C01; 10% difference) than PTH at week 22. With PTH, the increase in BMC was significantly higher in the distal tibia (C08-C10; 5–13% difference) than ML at weeks 20 and 22, with differences persisting following treatment withdrawal (week 24; C08-C10; 9 to 14%). By subregional analysis, greater osteogenic benefits of ML than PTH were observed in the mid-tibia postero-laterally, whereas PTH had greater benefit to the medial and more distal portions of the bone (Fig. S5, Supplementary Materials).

Compared with PTH, ML + PTH induced a greater increase in BMC at week 20 in the mid-tibia (C03-C04; 4–5% difference), extending proximally at week 22 (C01-C05; 6–17% difference) and persisting with treatment withdrawal (C01, C03-C06; 13 to 18%). With ML + PTH_alt_, the increase in BMC was higher in the mid-diaphysis at weeks 22 and 24 (C04-C05; 5 to 12% difference) than PTH; and higher in PTH than ML + PTH_alt_ distally (C09; 9% difference). Greater osteogenic benefits of combined treatments were observed in the proximal (C01-C05) postero-lateral compartments than PTH, whereas in the medial and anterior regions of the tibia similar or opposite (in C02-C03) results were found (Fig. S5, Supplementary Materials).

Compared with ML, a greater increase in BMC with ML + PTH was observed in both the proximal and distal tibia from week 20 (C01-C04, C10; 5 to 8% difference). Differences persisted at week 22 (C02-C04, C08-C10; 6 to 9% difference) and following treatment withdrawal (C02-C04, C08; 11 to 14%). At week 20, ML + PTH_alt_ induced a greater anabolic response than ML in the midshaft (C03-C04; 4 to 5% difference), but differences did not persist thereafter. ML + PTH induced a greater anabolic response in the proximal tibia than ML + PTH_alt_, but not until week 22 (C01-C04; 5–9% difference).

**Figure 8:**
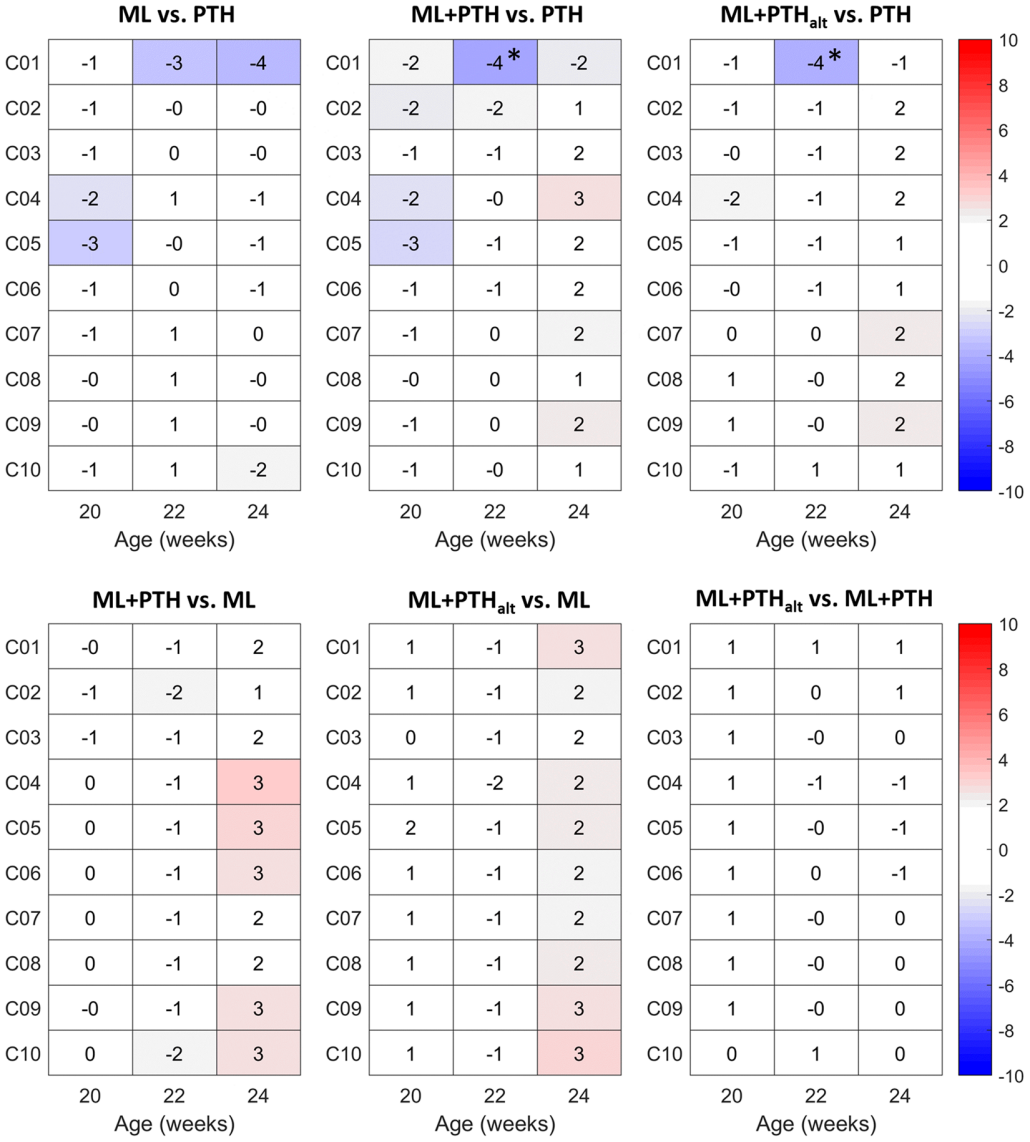
Longitudinal effects of PTH(1–34) and mechanical loading on tissue mineral density in 10 sections along the tibia length in ovariectomized C57BL/6 mice. Sections are C01, most proximal to C10, most distal. Values are reported as the relative percentage difference between two treatment groups (*g1* vs. *g2*), normalised for week 18 values of the latter group (*g2*). *p < 0.05, indicates statistically significant differences between groups (ANCOVA, adjusted for baseline values at week 18). Positive and negative values indicate greater increases in BMC in g1 and g2, respectively.

For TMD, the differences were small (less than 5%) and significantly differed only between PTH and combined treatment groups in the most proximal tibia region (C01; 4% lower in the combined treatments compared to PTH, Fig. 8).

## Discussion

In this study we quantified for the first time the longitudinal effects of PTH(1–34) and mechanical loading on bone morphometric and densitometric properties in an ovariectomised mouse model of osteoporosis. The results herein suggest a dominant effect of mechanical loading compared to injections of PTH, with increased and regionally-dependent benefits of combined treatments to the tibia cortical bone, but limited benefits for the trabecular bone.

PTH monotherapy had no significant anabolic benefit to metaphyseal trabecular bone consistent with neutral effects in intact mice^29,31,49^, but in contrast to increasing bone mass shown in OVX rodents elsewhere^17,19,20^. PTH inhibited further OVX-induced bone loss by trabecular thickening, a characteristic adaptive response^17,19,20^, in the presence of declining trabecular number. Interestingly, in 50% of the mice examined the trabecular changes were characterised by a small increase in Tb.BV/TV to week 20 and then bone loss thereafter (Supplementary Materials, Fig. S1), supporting a transient response to ongoing treatment described cross-sectionally^17^. Anabolic effects of PTH depend on its ability to stimulate osteoblast, osteocyte and osteoclast activities^50^. While typically favourable to osteoblastic activity, benefits may be compromised in mice with very low (<5%) baseline Tb.BV/TV as in C57BL/6 mice herein and reported elsewhere^17^. This is in line with the limited efficacy of PTH in the less trabecular rich femoral neck, relative to benefits in the lumbar vertebra shown clinically^51^. Brouwers *et al*.^19^ report, in OVX rats, strong positive interrelationships between baseline Tb.BV/TV and bony adaptations to PTH, though relationships were not confirmed by our current data (Tb.BV/TV_18_ vs. ∆Tb.BV/TV_18–22_; Spearman’s ρ=−0.657, p=0.156, see Supplementary Materials). In the midshaft, PTH treatment lead to an immediate increase (from week 18–20) in cortical thickness and bone area consistent with cross-sectional findings on OVX mice^17,20^, although osteogenic benefits, except in Tt.Ar, desisted thereafter. This finding in Ct.Th is contrary to the constant linear increase observed in OVX rats^19^, and in 19-months-old intact mice where PTH exacerbated age-related thinning of the cortical bone over time^29^. PTH had relatively homogeneous benefits along the bone length, increasing BMC with constant benefits to the mid- to distal tibia, and contrary to intact mice, where benefits propagated proximal to distally and in postero-medial sectors^31^.

Tibia loading had anabolic benefits to both the secondary trabecular and cortical bone shown previously in intact and orchidectomised mice^23,24,27,29^. In age-matched C57BL/6, OVX and age-related changes in trabecular metaphyseal bone are characterised by a significant decline in Tb.BV/TV (12–31% bone loss from 18- to 22-weeks-old)^36^. Whereas, bone adaptations after loading were characterised by a persistent increase in Tb.BV/TV (89% increase from week 18 to 22) due to trabecular thickening particularly in posterior regions (Fig. 3). In the mid-tibia, loading increased the cortical thickness and total cross-sectional area, and led to a constant increase in BMC in the proximal to mid-tibia, agreeable with cross-sectional^23,24,27,52^ and recent longitudinal findings^53^ in intact mice. Notably, the largest loading induced increase occurred posterior to laterally at the mid-shaft (Figs. 4 and 6), consistent with higher bone formation and decreased resorption processes at the periosteal surface of these sites documented elsewhere, and where the compressive strains are greatest under uniaxial load^54^. Compared with PTH, loading showed greater benefits to trabecular, but not cortical bone morphometry, except a higher cortical thickness two weeks after treatment withdrawal. PTH had greater benefit to distal BMC, whereas loading was more beneficial to the proximal and mid-tibia, consistent with heterogeneous strain distribution in this loading model^55^.

Both concurrent and alternating co-treatment had anabolic benefits to morphometric and densitometric properties of the mouse tibia. In general, owing to dominant loading effects as discussed above, combined treatment induced longitudinal adaptations in morphometric properties similar to loading alone, e.g. increased Tb.BV/TV with trabecular and cortical thickening. Interestingly, with co-treatment, PTH appeared to limit the osteogenic benefits of loading on the trabecular bone, confirming a possible antagonistic interaction on metaphyseal trabeculae observed cross-sectionally in intact 19-months-old C57BL/6^29^, though contrary to additive benefits on both appendicular or axial trabecular bone observed in 3–4 months-old intact^27^ and OVX C57BL/6 mice^15^. Combined treatments had increased benefits to cortical thickness than PTH alone, but not loading monotherapy which is consistent with short (2 weeks), but not with prolonged (3–6 weeks) tibia loading previously shown^27,29^. In BMC, PTH(1–34) enhanced loading effects in the proximal tibia, particularly in posterolateral regions that are subjected to higher compressive strain under controlled mechanical load, while conferring osteogenic benefits to the distal portion where mechanical effects were low. Compared with PTH, loading had increased benefits only to the proximal tibia and confirmed by site-specific analysis of cortical morphometry elsewhere^29^. Meanwhile, alternating PTH lead to a lower anabolic response at 22 weeks of age in the most proximal part of the tibia (significantly lower Tb.BV/TV and in C01-C04 BMC compared with ML + PTH).

An appropriate animal model of the human disease is recommended for preclinical testing of novel anti-osteoporotic treatment strategies. In C57BL/6, OVX-induced changes are characterized by rapid and persistent bone loss with concomitant reductions in circulating oestrogen^36^, the latter of which is not typical in aging rodents, though are cardinal features of human OP^56^. We selected skeletally mature, yet relatively young, mice to quantify adaptive response in absence of aging and related comorbidities that could confound the findings, though aging can affect the bone mechano-adaptation and responsiveness to co-therapy^29^, thus should be considered in future studies. With ovariectomy, our data highlights generally positive effects of combined bone anabolics to the cortical bone, but potentially antagonistic effects to trabecular bone in this mouse model. This variable response along the tibia, and often contrary to outcomes elsewhere, highlights the need for caution when extrapolating findings from young or old intact animals or otherwise positive anabolic benefits reported in axial bones^15^. Using comprehensive subregional assessment with longitudinal study design our results also provide meaningful additional information on bone’s dynamic response to treatments that can be underrepresented by standard morphometric analyses. For example, characterization of mid-shaft cortical morphometry failed to capture the increased and highly region-dependent benefits of combined PTH and loading that we demonstrate by BMC partitioning in quadrants along the bone length. This spatial analysis, applied to high-resolution *in vivo* microCT images, represents important methodological refinements by contributing to a substantial reduction in the number of animals used for preclinical assessment of novel anti-osteoporotic treatment strategies^32^. Further, the longitudinal data could provide invaluable information for mechanistic models of bone remodeling with anabolic therapies, e.g. references^57–59^.

There were limitations to this study. First, PTH(1–34) was administered approximately 2–3 hours following loading. While clinically, timing of the PTH(1–34) dose, e.g. morning than in evening, can enhance its efficacy^51^, the timing to optimise treatment synergies is yet to be resolved. Regardless, increased benefits were still shown and post-loading administration may be clinically relevant given drug side-effects, e.g. cramping and nausea/dizziness, which may be contraindications to exercise^8^. Second, the applied (12 N) load was matched across time-points and the intervention groups. Due to the PTH effects on cortical morphology, given at week 18 and one week before the first application of mechanical load, potential differences in local strains among the treatment groups may occur. Nevertheless, injections of PTH have shown not to significantly affect the cortical bone and to induce only small differences (7–9%) in BMC change in the proximal medial and posterior sectors^31^. Thus, the difference in local strain under the same axial load for the different groups of mice in week 19 should be minimal. Third, the *in vivo* study design precludes microCT scanning at smaller voxel size without increasing the radiation dose. Thus, we could not reasonably evaluate the effects of treatment on intra-cortical remodeling given that the mean cortical pore diameter in C57BL/6 mice is often less than the voxel size (i.e. <10 µm)^60^. Fourth, although sufficiently powered per our *a priori* sample size estimation, the heterogeneous response of the mice (see Supplementary Materials) may confound group trends and limit our ability to detect further significant intervention effects. However, the longitudinal design is advantageous to reduce risk of study bias while improving statistical power^32^. Finally, C57BL/6 mice, particularly following OVX have very low trabecular bone mass at treatment onset. Thus, in the tibia metaphysis there is often very few trabeculae on which to reliably assess treatment efficacy.

In conclusion, combining PTH(1–34) and tibia loading has increased, albeit highly regionally-dependent, benefits to the tibia cortical bone properties in ovariectomized mice, whereas co-treatment had lower osteogenic benefits on the trabecular bone than loading alone. While PTH(1–34) has relatively homogeneous benefits along the tibia length, loading increased BMC more focally in the mid-diaphysis and postero-laterally, which is subjected to higher stresses and strains under compressive loads. This data reinforces the need for comprehensive spatial analysis along the bone length when testing effects of novel treatment strategies.

## Supplementary information

Supplementary information.
