# Supplementary material for: PTH(1–34) treatment and/or mechanical loading have different osteogenic effects on the trabecular and cortical bone in the ovariectomized C57BL/6 mouse
Source: Sci Rep. 2020 Jun 1;10:8889. doi: 10.1038/s41598-020-65921-1 (PMC7264307; doi:10.1038/s41598-020-65921-1)
Supplement: Supplementary file 1 — Supplementary information. [file 41598_2020_65921_MOESM1_ESM.docx]

**PTH(1-34) treatment and/or mechanical loading have different osteogenic effects on the trabecular and cortical bone in the ovariectomized C57BL/6 mouse**

Bryant C Roberts^1,2,^*, Hector M Arredondo Carrera^1,3^, Sahand Zanjani-pour^1,2^, Maya Boudiffa^1,3^, Ning Wang^1,3^, Alison Gartland^1,3^ and Enrico Dall’Ara^1,2,3^

^1^Department of Oncology and Metabolism, University of Sheffield, Sheffield, United Kingdom

^2^INSIGNEO Institute for *in silico* Medicine, University of Sheffield, Sheffield, United Kingdom

^3^MRC Arthritis Research UK, Centre for Integrated Research into Musculoskeletal Ageing (CIMA), University of Sheffield, Sheffield, United Kingdom

***Corresponding author:**

Bryant C Roberts, PhD, Insigneo Institute for *in silico* Medicine, University of Sheffield, Pam Liversidge Building, S1 3JD, UK. Email: [b.c.roberts@sheffield.ac.uk](mailto:b.c.roberts@sheffield.ac.uk)

**Supplementary Materials**

**Computing *mean relative percentage difference* in bone densitometric properties**

BMC and TMD values in the 10 sections and 40 ROIs across the length of the tibia are reported as the mean relative percentage difference between two treatment groups [20]. First for each week (j), in each compartment, the mean changes in BMC relative to the week 18 values for Gp1 (∆Gp1_j_) and Gp2 (∆Gp2_j_) groups were calculated as below:

$${\boldsymbol{\Delta Gp}\boldsymbol{1}}_{\boldsymbol{j}}=\frac{\sum_{i=1}^{n1} ({BMC1}_{i,j}-{BMC1}_{i,18})}{n1}$$

$${\boldsymbol{\Delta Gp}\boldsymbol{2}}_{\boldsymbol{j}}=\frac{\sum_{i=1}^{n1} ({BMC2}_{i,j}-{BMC2}_{i,18})}{n1}$$

Where, *n1* and *n2* are the numbers of mice in Gp1 (BMC1) and Gp2 (BMC2) groups; *j* represents the week index and *i* represents the mouse number index.

Then for each week, in each compartment, the difference between the Gp1 (∆Gp1_j_) and Gp2 (∆Gp2_j_) groups normalized to the average BMC of the Gp1 mice at that week was calculated, in order to assess the effect of ovariectomy relative to the control group. This was defined as the mean relative percentage difference (δD%_j_) and was calculated as follows:

$$\boldsymbol{\delta D\%}_{\boldsymbol{j}}=({\Delta Gp2}_{j}-{\Delta Gp1}_{j})/{REF}_{j}\times100$$

Where,

$$\boldsymbol{REF}_{\boldsymbol{j}}=\frac{\sum_{i=1}^{n1} {BMC1}_{i,j}}{n1}$$

Similarly, mean relative percentage differences in TMD were computed as for BMC above.

**Individual patterns of trabecular and cortical bone changes in each treatment group**


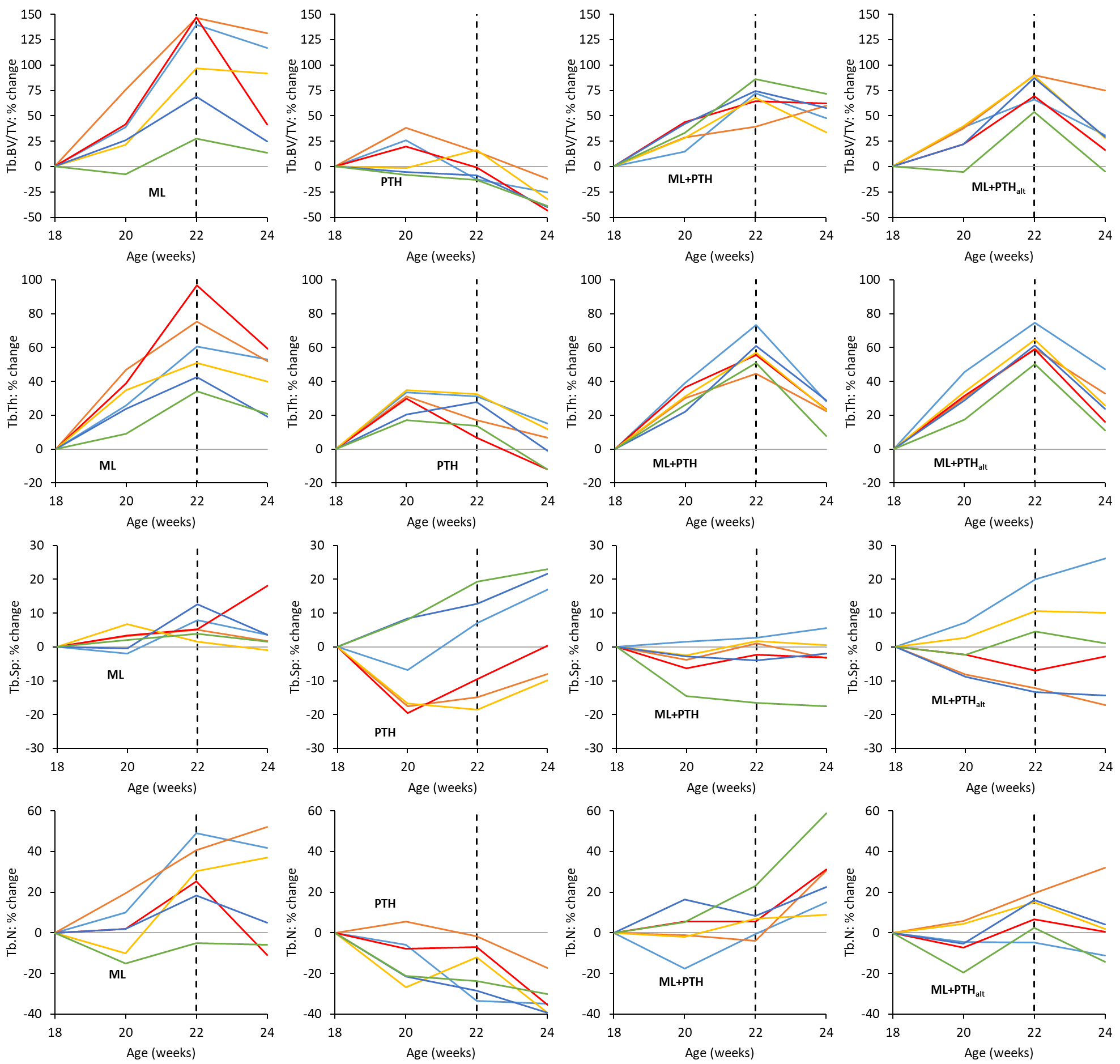


**Figure S1**: Percentage change, relative to treatment onset (week 18), in trabecular bone 3D morphometry in the proximal tibia metaphysis for individual ovariectomized mice following the individual or combined treatment with PTH(1-34) and mechanical loading. Ovariectomy was performed at 14 weeks old and treatment commenced at 18 weeks old and was withdrawn at 22 weeks old. Tb.BV/TV: bone volume fraction; Tb.Th: trabecular thickness; Tb.Sp: trabecular separation; Tb.N: trabecular number. Dashed line indicates time of treatment withdrawal.


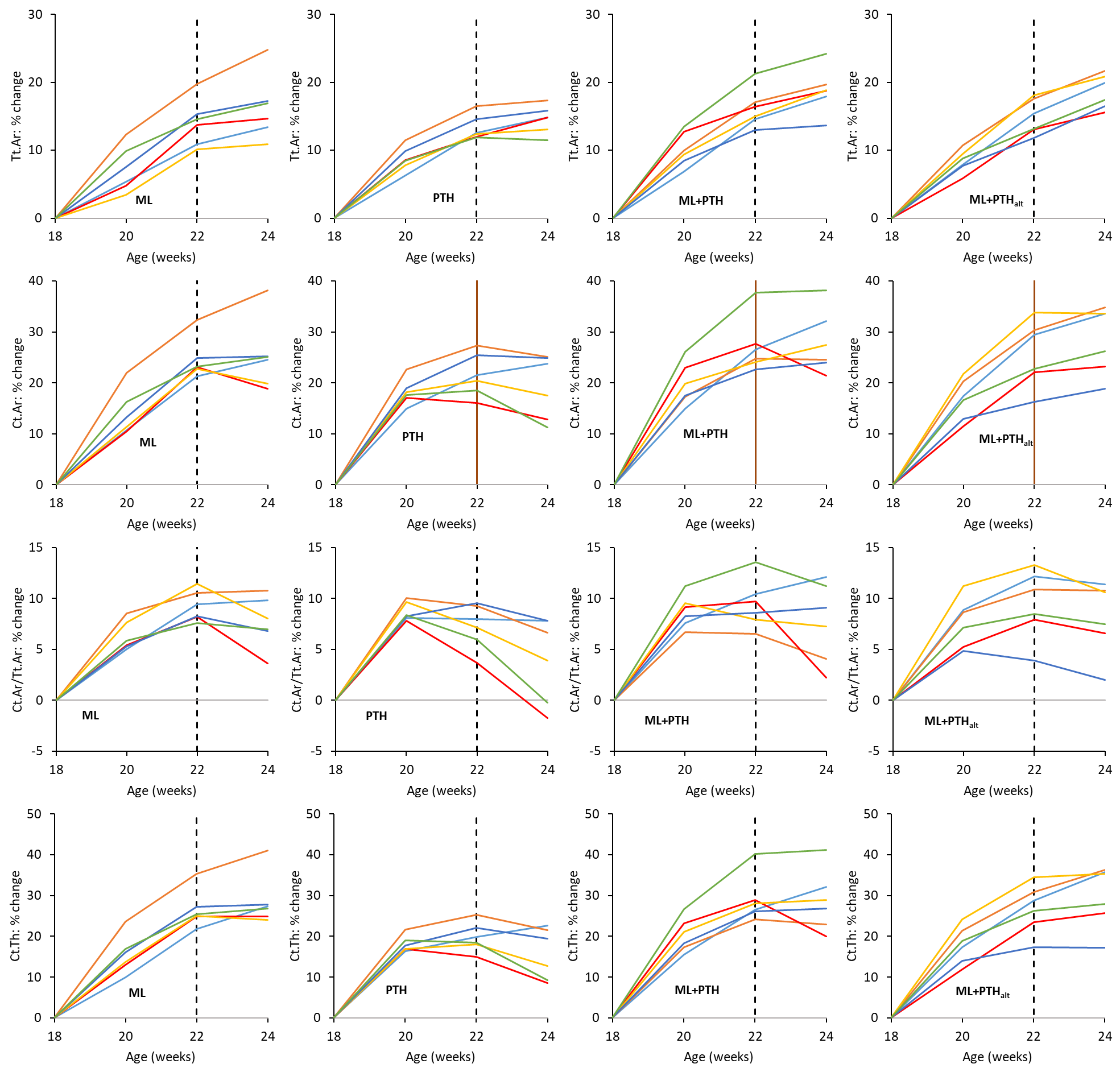


**Figure S2**: Percentage change, relative to treatment onset (week 18), in cortical bone 3D morphometry in the tibia midshaft for individual ovariectomized mice following the individual or combined treatment with PTH(1-34) and mechanical loading. Ovariectomy was performed at 14 weeks old and treatment commenced at 18 weeks old and was withdrawn at 22 weeks old. Tt.Ar: Total cross-sectional area; Ct.Ar: Total cortical area; Ct.Ar/Tt.Ar: Cortical area fraction; Ct.Th: Cortical thickness. Dashed line indicates time of treatment withdrawal.

**
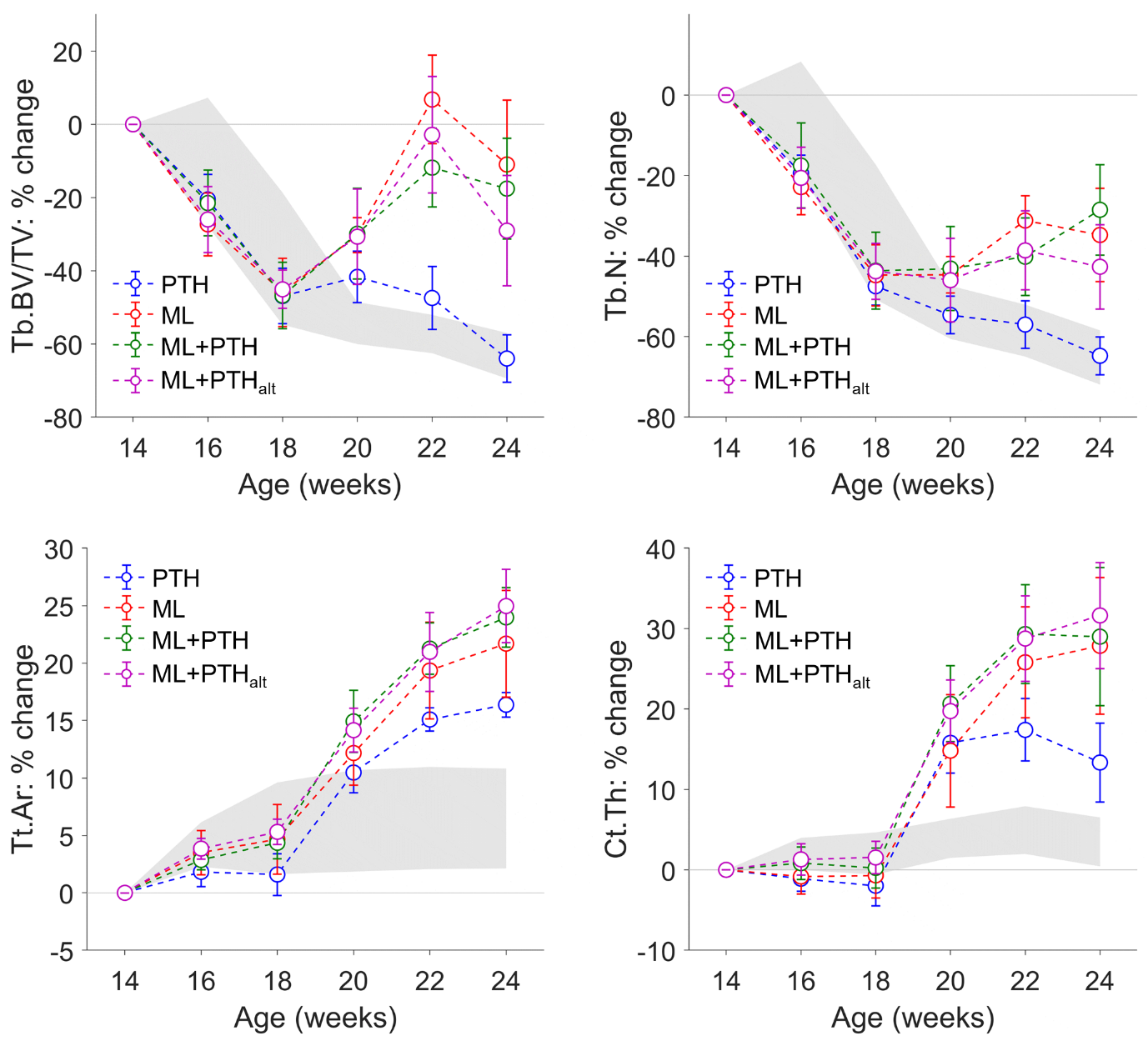
**

**Figure S3:** Mean percentage change (top) in Tb.BV/TV: trabecular bone volume fraction and Tb.N: trabecular number; (bottom) in Tt.Ar: cortical total area and Ct.Th: cortical thickness in the four treatment groups, relative to pre-ovariectomy (week 14) values. Treatment commenced at 18 weeks old and was withdrawn at 22 weeks old. Data from untreated ovariectomized mice from a previous study in our laboratory (Roberts et al.^36^) is shown in grey band (±1SD) highlighting the similar patterns of ovariectomy-induced bone adaptation among studies (until week 18) and marked response to treatment in the current study.

**Table S1** Tibial midshaft cortical bone moments of inertia and eccentricity over time for each treatment group with ovariectomy (values reported as mean ± standard deviation)

|  |  | **Age (weeks)** |  |  |  |  |  |
| --- | --- | --- | --- | --- | --- | --- | --- |
|  | Treatment | **14^⁋^** | **16** | **18*** | **20** | **22** | **24** |
| **I_max_ (mm^4^)** | PTH | 0.071 ± 0.003 | 0.073 ± 0.004 | 0.073 ± 0.003 | 0.093 ± 0.006 | 0.100 ± 0.007 | 0.101 ± 0.007 |
|  | ML | 0.066 ± 0.004 | 0.071 ± 0.003 | 0.073 ± 0.005 | 0.088 ± 0.009 | 0.105 ± 0.009 | 0.110 ± 0.010 |
|  | ML+PTH | 0.072 ± 0.003 | 0.076 ± 0.004 | 0.079 ± 0.005 | 0.102 ± 0.006 | 0.117 ± 0.005 | 0.123 ± 0.005 |
|  | ML+PTH_alt_ | 0.067 ± 0.007 | 0.073 ± 0.007 | 0.076 ± 0.008 | 0.094 ± 0.008 | 0.109 ± 0.008 | 0.118 ± 0.008 |
|  | OVX^‡^ | 0.054 ± 0.007 | 0.059 ± 0.005 | 0.061 ± 0.003 | 0.062 ± 0.004 | 0.062 ± 0.005 | 0.062 ± 0.006 |
|  | CTRL^‡^ | 0.053 ± 0.007 | 0.057 ± 0.006 | 0.059 ± 0.006 | 0.059 ± 0.006 | 0.061 ± 0.006 | 0.062 ± 0.006 |
|  | Time-effect:  PTH  ML  ML+PTH  ML+PTH_alt_ |  |  |  | (**<0.001**)  (**0.013**)  (**0.001**)  (<**0.001**) | (**<0.001**;**0.003**)  (**0.001**;**<0.001**)  (<**0.001**;**0.001**)  (<**0.001**;**0.003**) | (**<0.001**;1.000)  (**0.001**;0.146)  (<**0.001**;0.076)  (<**0.001**;**0.002**) |
| **I_min_ (mm^4^)** | PTH | 0.055 ± 0.004 | 0.056 ± 0.004 | 0.056 ± 0.004 | 0.068 ± 0.006 | 0.073 ± 0.006 | 0.073 ± 0.006 |
|  | ML | 0.049 ± 0.004 | 0.052 ± 0.004 | 0.053 ± 0.004 | 0.062 ± 0.004 | 0.068 ± 0.004 | 0.069 ± 0.006 |
|  | ML+PTH | 0.056 ± 0.005 | 0.059 ± 0.005 | 0.060 ± 0.006 | 0.075 ± 0.005 | 0.081 ± 0.004 | 0.082 ± 0.006 |
|  | ML+PTH_alt_ | 0.050 ± 0.005 | 0.054 ± 0.005 | 0.055 ± 0.005 | 0.066 ± 0.006 | 0.073 ± 0.006 | 0.076 ± 0.006 |
|  | OVX^‡^ | 0.043 ± 0.004 | 0.047 ± 0.004 | 0.048 ± 0.003 | 0.049 ± 0.004 | 0.050 ± 0.004 | 0.049 ± 0.005 |
|  | CTRL^‡^ | 0.041 ± 0.006 | 0.045 ± 0.006 | 0.047 ± 0.006 | 0.047 ± 0.007 | 0.048 ± 0.006 | 0.048 ± 0.006 |
|  | Time-effect:  PTH  ML  ML+PTH  ML+PTH_alt_ |  |  |  | (**0.001**)  (**0.004**)  (**<0.001**)  (**0.001**) | (**<0.001**;**0.013**)  (**<0.001**;**0.002**)  (**<0.001**;0.052)  (**<0.001**;**0.005**) | (**0.001**;1.000)  (**0.003**;1.000)  (**<0.001**;1.000)  (**<0.001**;**0.042**) |
| **J (mm^4^)** | PTH | 0.125 ± 0.007 | 0.130 ± 0.008 | 0.129 ± 0.007 | 0.160 ± 0.011 | 0.173 ± 0.012 | 0.174 ± 0.013 |
|  | ML | 0.115 ± 0.009 | 0.122 ± 0.006 | 0.126 ± 0.008 | 0.150 ± 0.013 | 0.173 ± 0.012 | 0.179 ± 0.015 |
|  | ML+PTH | 0.128 ± 0.007 | 0.135 ± 0.007 | 0.139 ± 0.007 | 0.188 ± 0.009 | 0.197 ± 0.004 | 0.205 ± 0.004 |
|  | ML+PTH_alt_ | 0.117 ± 0.011 | 0.127 ± 0.011 | 0.131 ± 0.012 | 0.161 ± 0.013 | 0.182 ± 0.012 | 0.193 ± 0.011 |
|  | OVX^‡^ | 0.097 ± 0.009 | 0.106 ± 0.008 | 0.109 ± 0.006 | 0.111 ± 0.007 | 0.112 ± 0.009 | 0.111 ± 0.010 |
|  | CTRL^‡^ | 0.094 ± 0.013 | 0.102 ± 0.012 | 0.105 ± 0.012 | 0.107 ± 0.013 | 0.110 ± 0.012 | 0.110 ± 0.012 |
|  | Time-effect:  PTH  ML  ML+PTH  ML+PTH_alt_ |  |  |  | (**<0.001**)  (**0.010**)  (**0.001**)  (**<0.001**) | (**<0.001**;**0.005**)  (**<0.001**;**<0.001**)  (**<0.001**;**0.003**)  (**<0.001**;**0.002**) | (**<0.001**;1.000)  (**0.001**;0.313)  (**<0.001**;0.077)  (**<0.001**;**0.003**) |
| **Ecc (*unitless*)** | PTH | 0.471 ± 0.030 | 0.473 ± 0.031 | 0.477 ± 0.038 | 0.517 ± 0.034 | 0.522 ± 0.028 | 0.525 ± 0.028 |
|  | ML | 0.504 ± 0.023 | 0.512 ± 0.031 | 0.514 ± 0.034 | 0.546 ± 0.036 | 0.583 ± 0.035 | 0.607 ± 0.021 |
|  | ML+PTH | 0.464 ± 0.057 | 0.469 ± 0.060 | 0.476 ± 0.070 | 0.512 ± 0.057 | 0.551 ± 0.057 | 0.565 ± 0.064 |
|  | ML+PTH_alt_ | 0.489 ± 0.054 | 0.502 ± 0.054 | 0.509 ± 0.051 | 0.541 ± 0.049 | 0.572 ± 0.046 | 0.591 ± 0.040 |
|  | OVX^‡^ | 0.444 ± 0.025 | 0.450 ± 0.027 | 0.448 ± 0.041 | 0.444 ± 0.047 | 0.442 ± 0.036 | 0.454 ± 0.046 |
|  | CTRL^‡^ | 0.466 ± 0.041 | 0.466 ± 0.043 | 0.453 ± 0.051 | 0.451 ± 0.061 | 0.460 ± 0.032 | 0.472 ± 0.037 |
|  | Time-effect:  PTH  ML  ML+PTH  ML+PTH_alt_ |  |  |  | (**0.021**)  (**0.041**)  (**0.047**)  (**0.022**) | (0.060;1.000)  (**<0.001**;<**0.001**)  (**0.008**;**0.002**)  (**<0.001**;**0.008**) | (0.052;1.000)  (**0.001**;0.147)  (**0.018**;0.991)  (**<0.001**;**0.011**) |

I_max_: maximum moment of inertia, I_min_: minimum moment of inertia, J: Polar moment of inertia, Ecc: eccentricity. The p-values for a “time-effect” are reported in parentheses as (comparison to baseline (week 18) values; comparison previous time point). Bold values indicate a statistically significant difference between time points. Superscript: **^⁋^**Ovariectomy was performed at week 14; *treatment commenced, per Fig. 1(a), at the beginning of week 18 and was withdrawn at the end of week 22. ^‡^Properties of untreated ovariectomized mice (group “OVX”) and intact controls (“CTRL”) from Roberts et al.^36^ are reported for comparison of trends in bone adaptation.


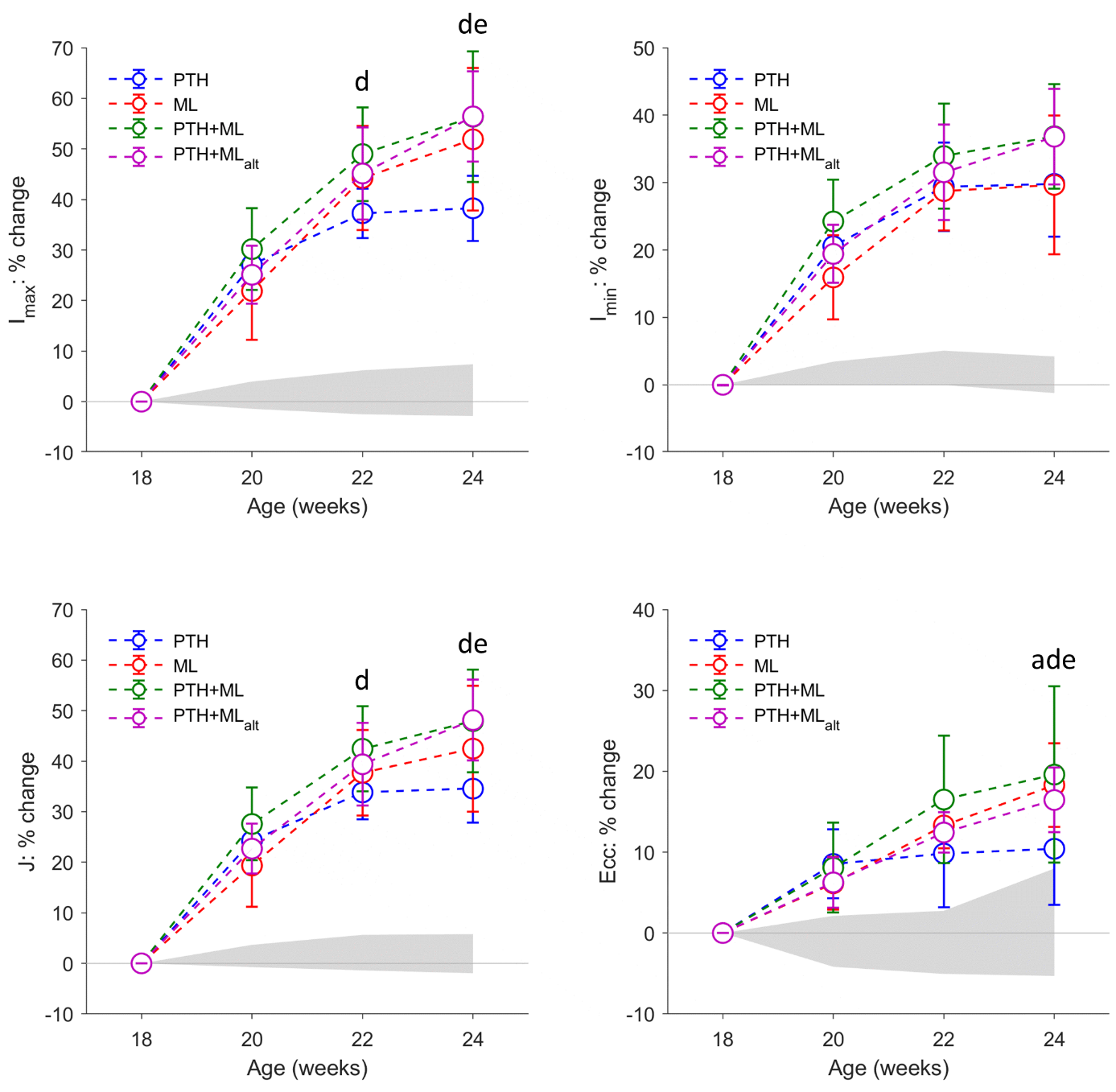


**Figure S4:** Mean percentage change, relative to week 18 values, in cortical midshaft moments of inertia and eccentricity. Statistically significant differences between groups are noted (p<0.05; ANCOVA, adjusted for week 18 values with post hoc Bonferroni adjustment): ^a^PTH vs. ML; ^d^PTH vs. ML+PTH; ^e^PTH vs. ML+PTH_alt_. Data from untreated ovariectomized mice from a previous study in our laboratory (Roberts et al.^36^) is shown in grey bands (±1SD) highlighting the marked treatment effects of individual and combined treatment with PTH(1-34) and loading the bone properties in C57BL/6 mice.

**
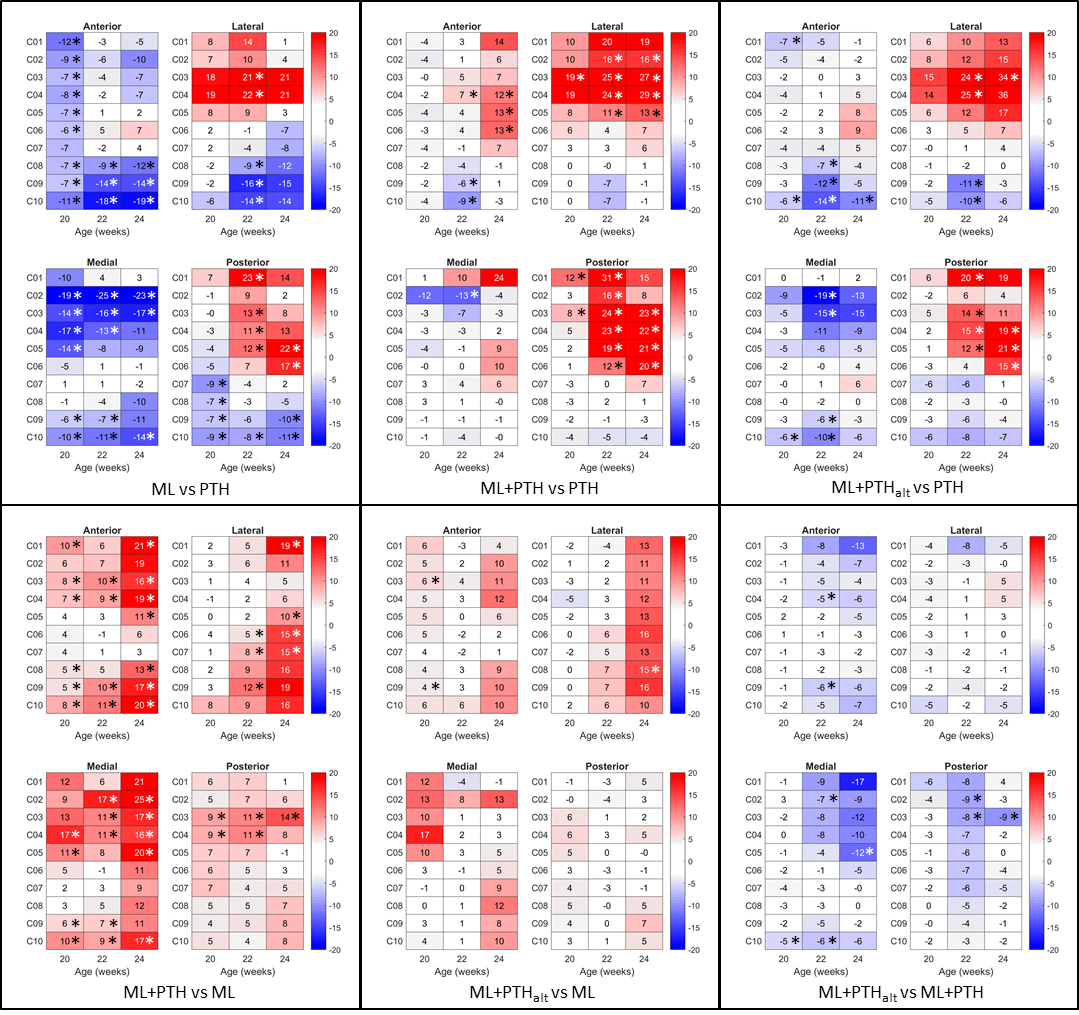
**

**Figure S5:** Longitudinal effects of PTH(1-34) and mechanical loading on the bone mineral content in four anatomical quadrants (lateral, anterior, medial and posterior) at ten sections along the tibia length of ovariectomized mice. Ovariectomy was performed at 14 weeks old and treatment commenced and then was withdrawn at weeks 18 and 22 respectively. *p<0.05, statistically significant differences between treatment groups

**
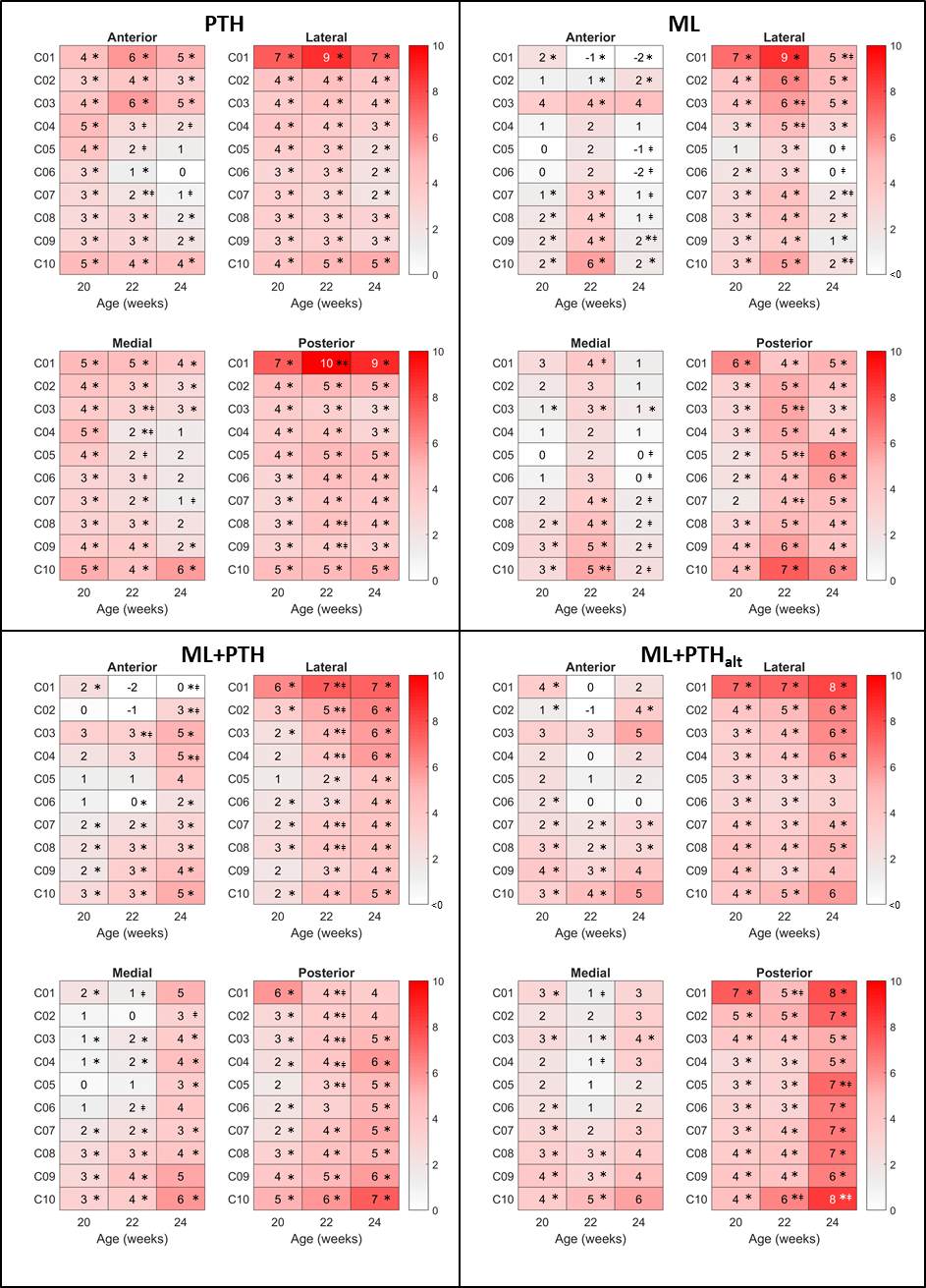
 Figure S6**: Mean percentage change, relative to week 18 values, in tissue mineral density in 40 subregions of interest along 80% of the tibia length in the four treatment groups. Ovariectomy was performed at 14 weeks old and treatment commenced at 18 weeks old and was withdrawn at 22 weeks old. *Statistically significant difference compared with week 18 and ^‡^between sequential timepoints (p<0.05; ANOVA with post hoc pairwise comparisons).

**Table S2** Relationships between baseline trabecular BV/TV (Tb.BV/TV_18_) and change in trabecular BV/TV from treatment onset to withdrawal (∆Tb.BV/TV_18-22_)

| Group | Spearman’s rho | p-value |
| --- | --- | --- |
| PTH | -0.657 | 0.156 |
| ML | -0.771 | 0.072 |
| ML+PTH | -0.143 | 0.787 |
| ML+PTH_alt_ | -0.314 | 0.544 |
